# Revolutionizing Cardiology through Artificial Intelligence—Big Data from Proactive Prevention to Precise Diagnostics and Cutting-Edge Treatment—A Comprehensive Review of the Past 5 Years

**DOI:** 10.3390/diagnostics14111103

**Published:** 2024-05-26

**Authors:** Elena Stamate, Alin-Ionut Piraianu, Oana Roxana Ciobotaru, Rodica Crassas, Oana Duca, Ana Fulga, Ionica Grigore, Vlad Vintila, Iuliu Fulga, Octavian Catalin Ciobotaru

**Affiliations:** 1 Department of Cardiology, Emergency University Hospital of Bucharest, 050098 Bucharest, Romania; elena.stamate94@yahoo.com (E.S.); vladvintila2005@yahoo.com (V.V.); 2 Faculty of Medicine and Pharmacy, University “Dunarea de Jos” of Galati, 35 AI Cuza Street, 800010 Galati, Romania; oanam.duca@gmail.com (O.D.); ana.fulgaa@yahoo.com (A.F.); ionicagrigore2004@yahoo.com (I.G.); fulgaiuliu@yahoo.com (I.F.); coctavian72@gmail.com (O.C.C.); 3 Railway Hospital Galati, 800223 Galati, Romania; 4 Emergency County Hospital Braila, 810325 Braila, Romania; rodica.crassas@gmail.com; 5 Saint Apostle Andrew Emergency County Clinical Hospital, 177 Brailei Street, 800578 Galati, Romania; 6 Clinical Department of Cardio-Thoracic Pathology, University of Medicine and Pharmacy “Carol Davila” Bucharest, 37 Dionisie Lupu Street, 4192910 Bucharest, Romania

**Keywords:** artificial intelligence, machine learning, deep learning, cardiology, valvular disease, arithmology

## Abstract

Background: Artificial intelligence (AI) can radically change almost every aspect of the human experience. In the medical field, there are numerous applications of AI and subsequently, in a relatively short time, significant progress has been made. Cardiology is not immune to this trend, this fact being supported by the exponential increase in the number of publications in which the algorithms play an important role in data analysis, pattern discovery, identification of anomalies, and therapeutic decision making. Furthermore, with technological development, there have appeared new models of machine learning (ML) and deep learning (DP) that are capable of exploring various applications of AI in cardiology, including areas such as prevention, cardiovascular imaging, electrophysiology, interventional cardiology, and many others. In this sense, the present article aims to provide a general vision of the current state of AI use in cardiology. Results: We identified and included a subset of 200 papers directly relevant to the current research covering a wide range of applications. Thus, this paper presents AI applications in cardiovascular imaging, arithmology, clinical or emergency cardiology, cardiovascular prevention, and interventional procedures in a summarized manner. Recent studies from the highly scientific literature demonstrate the feasibility and advantages of using AI in different branches of cardiology. Conclusions: The integration of AI in cardiology offers promising perspectives for increasing accuracy by decreasing the error rate and increasing efficiency in cardiovascular practice. From predicting the risk of sudden death or the ability to respond to cardiac resynchronization therapy to the diagnosis of pulmonary embolism or the early detection of valvular diseases, AI algorithms have shown their potential to mitigate human error and provide feasible solutions. At the same time, limits imposed by the small samples studied are highlighted alongside the challenges presented by ethical implementation; these relate to legal implications regarding responsibility and decision making processes, ensuring patient confidentiality and data security. All these constitute future research directions that will allow the integration of AI in the progress of cardiology.

## 1. Introduction 

Artificial intelligence (AI) has penetrated all aspects of life and has recently stood out through the development of deep learning models that can generate almost anything with minimal human intervention. However, among all fields of activity, medicine has emerged as a particularly significant one, with great potential for development [1]. Among all specialties wherein AI has found its place through clinical applications, cardiology holds a leading position. According to the World Health Organization, the main cause of death globally, accounting for approximately a third of annual deaths, is cardiovascular disease [2].

When it comes to healthcare, a paradigm shift has been triggered with the integration of AI into various medical disciplines, including cardiology. Therefore, AI could revolutionize cardiology by transforming the way cardiovascular diseases are prevented, diagnosed, and treated. It includes different methods that allow machines to mimic human behaviors such as learning, reasoning, problem solving, perception, and decision making. In cardiology, all of these can lead to providing accurate predictions and personalized information and can even identify patterns [3]. Artificial intelligence techniques have shown their power to enhance progress in the management of atherosclerotic cardiovascular disease, heart failure, atrial fibrillation, pulmonary embolism, hypertension, pulmonary hypertension, valvular heart diseases, cardiomyopathies, congenital heart diseases, and more [4]. However, expertise in pathophysiology and patient clinical knowledge will not be replaced, the human element remaining vital in the medical process, with physicians ultimately deciding where to apply and how to interpret the data provided by AI [5]. The main advantage of AI lies in its ability to analyze a large database in a short time and provide targeted information tailored to each category of patients [6,7]. In addition, deep learning algorithms, which are the most commonly applied AI subcategory in medicine at this moment [8], allow for the partial elimination of human error from the medical process by reducing human involvement, correcting clinician errors, and preventing misdiagnosis, which constitutes another advantage of AI in healthcare [9,10].

**Figure 1:**
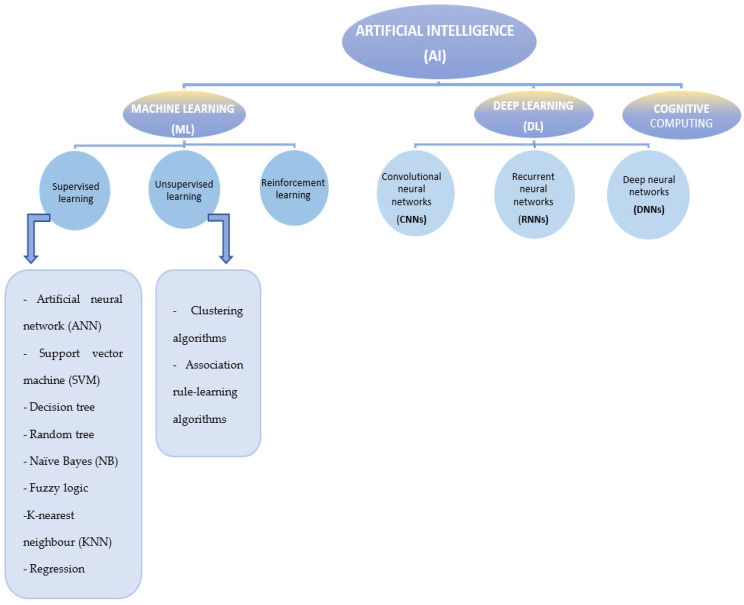
Illustration of the AI subtypes. Created based on information from [4,13].

In the healthcare field, artificial intelligence has the potential to open up new perspectives through personalized approaches to each patient. Thus, integrating AI into routine medical practice supports medical activity, can increase the success rate in treating cardiovascular diseases, and can improve the quality of medical care whilst recognizing the limits of AI and not minimizing its ethical and legal issues [11]. This summary aims to provide a synthesis of the application of AI in cardiology for easier understanding of AI and to support the use of AI in the daily practice of the cardiologist. The relationship between AI and its subdisciplines—machine learning (ML), deep learning (DL), and cognitive computing—is visually represented in Figure 1. In essence, both machine learning and deep learning fall under the umbrella of artificial intelligence. Machine learning, as an innovative field, enables systems to adapt and improve with minimal human intervention. Deep learning, in turn, is a subset of machine learning that focuses on artificial neural networks to mimic the learning process of the human brain. Deep learning is an evolution of machine learning [12]. Additionally, Table 1 briefly exemplifies the most relevant concepts of AI tools.

## 2. Literature Review

### 2.1. Methodology

We conducted a comprehensive review of current literature including original articles that studied various clinical applications of AI in cardiology. We performed extensive searches on PubMed, Google Scholar, ScienceDirect, Elsevier, Scopus, Web of Science, and Cochrane databases to identify relevant manuscripts. We used three sets of keywords to recognize terms from the title, abstract, and keywords of the studies: (i) the first set of keywords included terms associated with artificial intelligence, such as “artificial intelligence”, “deep learning”, “machine learning”, “prediction”, “diagnosis”, “screening”, “treatment”, and “prognosis”. However, studies using these methodologies are likely to incorporate terms such as “artificial intelligence” or “machine learning” in their abstracts or keywords. (ii) The second set of keywords included domains associated with applicability in clinical practice. Thus, compound searches were performed using the terms “artificial intelligence” combined with a chosen cardiology domain: “arithmology”, “cardiac imaging”, “ischemic heart disease”, “valvular disease”, “heart failure”, “congenital diseases”, “hypertension”, and more. We restricted our search to papers published in English in the last 5 years, between 2020 and 2024; additionally, textbooks on AI were consulted, and we found more than 973 relevant manuscripts. 

We removed duplicate articles and then conducted a detailed evaluation of abstracts and titles to determine their suitability for inclusion. The selection criteria focused on studies examining the application of artificial intelligence in various branches of cardiology. Subsequently, we systematically applied selection criteria to evaluate the studies. Studies were assessed based on the following criteria: (1) journal, (2) publication date, (3) study design, (4) analysis methods, (5) results, and (6) conclusions. We initially screened abstracts and eliminated studies not written in English. To ensure data quality, we paid close attention to specific aspects regarding the comprehensive evaluation of studies meeting the inclusion criteria, such as justification, method design, results, discussions, conclusions, and any signs of methodological bias or interpretation of data that could have a negative impact on the results of the studies reviewed.

Essentially, the inclusion criteria were as follows:

1. Studies examining the application of artificial intelligence in various branches of cardiology, such as arrhythmology, emergency cardiology, cardiomyopathies, cardiovascular imaging, congenital cardiovascular disease, electrocardiography, heart failure, heart transplantation, hypertension, pulmonary hypertension, infective endocarditis, ischemic heart disease, pericardial disease, peripheral heart disease, thromboembolic disease, and valvular diseases (this is a broad selection criterion focusing on the theme of studies relevant to the proposed review and represents the main topic of the article);
2. Publications in English;
3. Published within the last 5 years, between 2020 and 2024 (this temporal restriction ensures the timeliness and relevance of the information included in the review);
4. Patient batches that included both adults and children (this criterion ensured a larger batch of studies covering cardiology);
5. Studies in the form of an academic journal article.

Exclusion criteria:

1. Articles in languages other than English;
2. Retracted studies (eliminating retracted studies is essential to maintain the integrity and credibility of this review);
3. Applications of artificial intelligence regarding technical functionality data of algorithms (excluding these studies may be justified to focus on the practical and clinical application of artificial intelligence in cardiology, rather than the technical aspects of algorithms);
4. Studies in the form of posters, short papers, or only abstracts;
5. Duplicate studies;
6. Studies with a title and abstract that do not match the review topic.

The limitations of the review process included variations in methodologies among the included studies and potential publication biases. Additionally, the rapidly evolving nature of AI technologies in healthcare may introduce limitations in capturing the latest developments. Additionally, limitations of the study are issues related to ethical implementation and legal issues regarding accountability and decision making; still, in small batches of patients, the susceptibility model is considered a “black box” and standardization of the method. These may be future research directions in AI [34].

### 2.2. Results 

**Figure 2:**
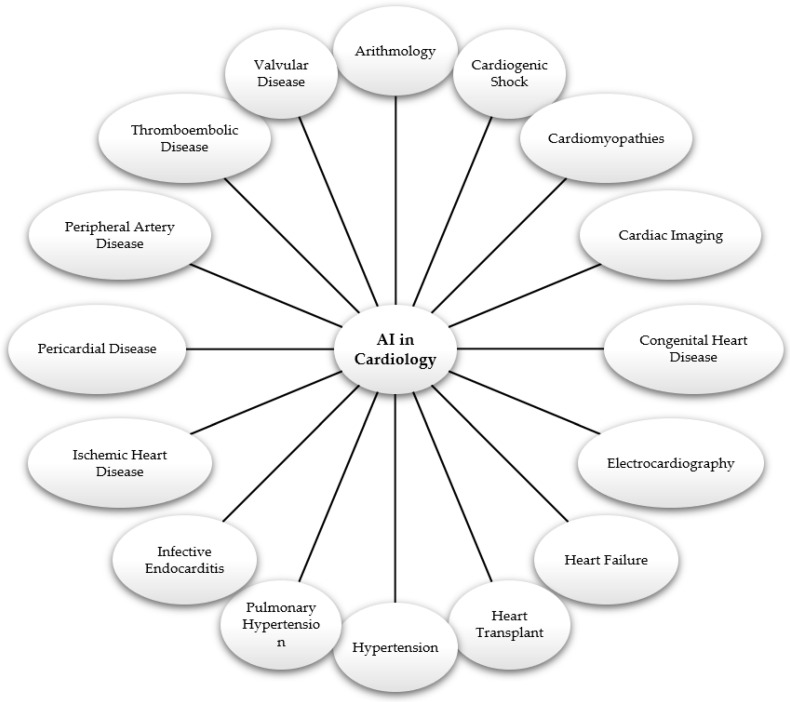
Application areas of AI in cardiology—main points of the review.

After a thorough review and assessment of the 665 articles, we identified and included a subset of 200 papers that were directly relevant to our research, including 5 on arithmology, 10 on cardiogenic shock, 21 on cardiomyopathies, 18 on cardiac imaging, 6 on congenital heart disease, 11 on electrocardiography, 13 on heart failure, 14 on heart transplant, 14 on hypertension, 25 on pulmonary hypertension, 3 on infective endocarditis, 21 on ischemic heart disease, 5 on pericardial disease, 8 on peripheral artery disease, 12 on thromboembolic disease, and 14 on valvular disease. These areas of application of AI in cardiology are represented in Figure 2. These selected studies provided valuable insights into the use and impact of AI in cardiology, forming the basis of our review. 

The 200 scientific articles that analyze the current applications of artificial intelligence in cardiology, as well as future research perspectives, are schematically summarized in Table 2.

#### 2.2.1. AI in Arrhythmias 

One of the most common arrhythmias in adults is atrial fibrillation (AF), with an estimated prevalence ranging from 2% to 4% [230]. Because one-third of people with arrhythmia are asymptomatic, diagnosing AF can be challenging. AF often presents intermittently, referred to as paroxysmal atrial fibrillation (AF), which is often undiagnosed, resulting in significant mortality and morbidity. Strategies for detecting AF include serial electrocardiography (ECG), event monitors, long-term outpatient monitoring using wearable continuous ECGs, in-hospital monitoring, or implantable cardiac monitors. However, AF detection rates remain low, between 5% and 20%, despite these measures. Predicting the timing of the onset of AF could improve the treatment of this condition, especially since AF is expected to affect more than 12 million people in the U.S. by 2030. Thus, there is a need to identify innovative and cost-effective techniques, especially in terms of cost, to help clinicians better treat this disease [36,37]. 

The ECG has been analyzed since the 1970s when ventricular repolarization abnormalities were analyzed by an AI-based model, which finally showed a high correlation with serum potassium levels [231]. In 2023, the way AF prediction and detection are evolving with the availability of new predictive tools was well described in a review carried out by Martínez-Sellés, M. et Marina-Breysse, M [232]. 

In this review, the authors showed how an AI-enabled ECG acquired during normal sinus rhythm allows point-of-care identification of people with AF. Other authors have explored AF prediction using mobile sinus rhythm electrocardiograms (mECG) and demonstrated that neural networks can predict AF development using mECG data in sinus rhythm. They concluded that mECG data could lower barriers to the implementation of AI-based AF event prediction systems in the modern healthcare environment due to their cost-effectiveness, availability, and scalability [37]. 

A study of 2530 patients showed that the CNN model had better predictive performance than other current predictive models in effectively predicting the risk of postoperative recurrence in patients with paroxysmal atrial fibrillation by identifying 12-lead ECG characteristics before catheter ablation [38]. 

In January 2024, a paper was published in JAHA, developing robust deep learning algorithms for automated ECG detection of postoperative AF and its burden using both atrial and surface ECGs. This finding has an important impact on the subsequent management of patients with newly diagnosed AF [233]. Overall, ML models show promise for detecting AF in a stroke population for secondary stroke prevention and for accurately predicting AF in a healthy population for primary prevention. 

While previous authors analyze models to predict the risk of FA or to detect FA in at-risk populations, other authors focus on the applicability of these models. Kawamura, Y. concludes in a review that implementation in the real world of AF prediction models requires validation studies and the development of points that would facilitate transparency through reducing potential systemic biases and improving generalizability [234]. 

However, another review in 2024, which included 14 studies, showed that AI is effective for detecting AF from ECGs. Among DL algorithms, convolutional neural networks (CNNs) demonstrate superior performance in AF detection compared to traditional machine learning (TML) algorithms. Diagnosing AF earlier can integrate ML algorithms that can help wearable devices [235].

#### 2.2.2. AI in Cardiogenic Shock

CS is a pathology represented by low cardiac output causing hypoperfusion of the target organs. CS causes very high short-term mortality of up to 50%. Observational studies have shown that early recognition, protocol management, optimal triage, and risk stratification in hospitals equipped with technology and well-trained staff have led to much better outcomes in the management of CS [236].

In January 2024 Raheem A. et al. published a retrospective study, which looked at 97,333 patients, in which they described a new, much more detailed way to predict MACE, in-hospital mortality (up to 30 days) from all causes, and cardiac arrest. They used AI and a systemic grid technique in an ANN to robustly analyze the performance of the ANN model compared to RF and LR classifiers and the commonly used Emergency Severity Index (ESI). The authors created a predictive model, based on emergency room presentation criteria, that would make it easier for emergency physicians to triage patients with cardiovascular symptoms. They demonstrated that ANN with systematic grid search predicted MACE, cardiac arrest, and 30-day in-hospital mortality in triaging patients with cardiovascular symptoms with high accuracy, unlike LR and RF models. Their predictive model could therefore help emergency physicians make timely triage choices for patients with cardiovascular symptoms by classifying and prioritizing patients in the early phase based on triage presentation criteria [237]. On the other hand, another study analyzing 2282 STEMI patients demonstrated that for predicting cardiogenic shock in STEMI patients, the linear LASSO model showed superiority over LR, SVM, and XGBoos. In patients with AMI, CS is the most common cause of in-hospital death, accounting for 5–10% of patients [41]. In most of the studies analyzed, the repeatable limitations include retrospective studies conducted on target populations. Future prospective studies are therefore needed, including populations from more than one center [42,47,48].

#### 2.2.3. AI in Cardiomyopathy

Numerous studies have analyzed electrocardiograms using artificial intelligence and have proven their usefulness in detecting cardiomyopathies and more [51,54,55,64]. ECG analysis using AI has shown its usefulness in both adult [51,55] and pediatric hypertrophic cardiomyopathy [54] in the detection of cardiac amyloidosis [64]. Haimovich, J. S. et al. published a study in 2023 that included 93,138 adult patients and concluded that models based on ECG analysis, LVH-NET, and its single-lead versions may be useful in the clinic for screening patients with left ventricular hypertrophy as well as rare diseases such as hypertrophic cardiomyopathy and cardiac amyloidosis [51]. Another previously mentioned study looked at ECGs from 300 children and adolescents under the age of 18 and showed their usefulness in detecting pediatric hypertrophic cardiomyopathy. In 2023, Harmon D.M. et al. studied 676 patients who were evaluated at the Mayo Clinic and diagnosed with AL or ATTR cardiac amyloidosis (CA). The authors demonstrated that AI-ECG achieved very high performance for detecting CA in terms of sex, race, age, and amyloid subtype. On the other hand, the AI-ECG demonstrated lower performance for patients with LBBB [64]. Echocardiography is another tool that with the help of AI can bring closer diagnoses of CA. Cotella J. I et al. studied 51 patients who calculated FEVS and GLS using AI, and they proved that there were no significant differences in manual and automated LVEF and GLS values either pre-CA or at diagnosis. This would allow for a faster evaluation of CA patients [65].

Zhang X. et al. showed in a retrospective analysis of 289 patients that ultrasonic imaging omics and a machine learning model can provide an excellent and non-invasive diagnostic tool for clinical practice for distinguishing CA from non-CA. For left ventricular strain, the machine learning model was slightly better than conventional echocardiography [66]. Another study, which analyzed 128 patients with ATTR-CA using AI, concluded that the ANN model estimated the risk of death or transplantation in patients with ATTR cm with better accuracy compared to traditional risk models [67].

Takotsubo (TTS) cardiomyopathy is another cardiomyopathy in which the application of AI has found a place. Takotsubo cardiomyopathy (transient apical ballooning syndrome or broken heart syndrome) is a form of non-ischemic cardiomyopathy. TTS predominantly affects women and is a regional left ventricular systolic dysfunction; it is transient but occurs without significant coronary artery disease on angiography [238]. Echocardiography, coronary angiography, left ventriculogram, and cardiac magnetic resonance imaging (CMR) are used to diagnose TTS. As a clinical entity of acute transient heart failure, its general management is conventional heart failure therapy if the patient does not show hemodynamic instability or mechanical complications [239].

For patients who are not eligible for gadolinium contrast CMR, the diagnosis of takotsubo cardiomyopathy remains difficult without invasive investigation. One study that analyzed non-contrast CMR images and demographic data of cardiac arrest patients using AI found a model that offers good accuracy in predicting patients with Takotsubo (TTS) cardiomyopathy [58]. Another study, which looked at 3284 patients with TTS, showed that an ML-based approach identified patients at risk of a poor short-term prognosis in the hospital. The Inter-TAK-ML model has shown its usefulness for predicting in-hospital death in patients with TTS [59].

Another rare genetic cardiomyopathy is Fabry disease (FD). It has multisystem involvement and a reported but possibly underestimated annual incidence of 1 in 100,000. Many cases go undiagnosed because there is a large age gap between the age at which the first symptoms appear and the age at which it is diagnosed; this is 13 and 32 for women and 9 and 23 for men [240]. Symptoms of onset include neuropathic pain, recurrent fever, ophthalmic problems, sweating disorders, typical skin changes, gastrointestinal symptoms, heat/cold intolerance, and otolaryngological problems. However, the most serious problems induced by FD include cerebrovascular cardiovascular events and cardiac dysfunction, cardiovascular and cerebrovascular events, and chronic kidney disease, usually with proteinuria. Michalski A.A. et al. evaluated risk factors among patients who may suffer from FD and demonstrated that an NLP tool approach increased diagnostic effectiveness and improved prognosis and quality of life for patients with Fabry disease. The method also recognized its limitations, which consisted of the need for prospective studies, the small sample of patients diagnosed with FD, the analyzed risk factors, and the implemented NLP algorithm which requires further development to improve its accuracy [68]. In patients with FD, cardiac arrhythmias are common, but individual risk varies widely. Among the most common arrhythmias are ventricular tachycardia and atrial fibrillation. Jefferies J. et al. conducted a study on 5904 patients with FD in which AI-machine learning models were applied and demonstrated strong performance in estimating the risk of adverse outcomes. This discovery could be useful in clinical practice where it would be used to reduce patients’ adverse outcomes and improve their management [69].

#### 2.2.4. AI in Cardiovascular Imaging

Artificial intelligence (AI) is spreading into every facet of cardiac imaging, from studies to prognostication and personalized risk prediction for each patient. The Food and Drug Administration (FDA) has approved approximately 300 artificial intelligence devices in the combined fields of radiology and cardiovascular, and this number continues to grow. Cardiac magnetic resonance imaging (CMR), echocardiography, and coronary computed tomography angiography (CCTA) derive significant benefits from AI-based solutions. AI has numerous advantages in cardiovascular imaging, from the possibilities of increasing efficiency to reducing inter-observer and intra-observer variability and also reducing human error and reader variability [241].

Artificial intelligence has also found its place in cardiovascular imaging. Using machine learning methods and radiomic features from delayed enhancement CT (CT-DE), myocardial scarring has been identified with good accuracy compared to cardiac magnetic resonance imaging (MRI) with late gadolinium enhancement (LGE) (MRI-LGE), which is the gold standard [73]. Additionally, using CMR radiomic features, other authors in another study created a predictive model to classify patients with hypertrophic cardiomyopathy (HCM) and dilated cardiomyopathy (DCM) [71]. A retrospective study of 303 patients analyzing coronary CT, fractional flow reserve (FFR), and quantitative coronary angiography (QCA) data demonstrated that AI has its place in coronary CT. The authors demonstrated rapid and accurate identification of major stenoses, superimposable with coronary angiography [76]. Zhang R et al. published a retrospective study in which they analyzed 599 patients who underwent myocardial perfusion imaging (MPI). Image analysis was performed using hybrid SPECT-CT systems. The authors aimed to validate and develop an AI (artificial intelligence) aid method applied in myocardial perfusion imaging (MPI) to help clinicians differentiate ischemia in coronary artery disease. They demonstrated the high predictive value and very good efficacy of this system and therefore found a tool to help radiologists in their clinical practice [80]. Another study published in the European Heart Journal of 2827 patients that analyzed echocardiographic images using AI demonstrated increased accuracy in diagnosing left atrial thrombosis (LAT). This finding guides clinicians in the management of patients on oral anticoagulant (OAC) therapy in deciding on transesophageal ultrasound (TOE) [82]. 

Another large study published in the Jama analyzed transthoracic ultrasounds of 224 patients with Takotsubo and 224 patients with AMI to differentiate between the two diseases. The authors demonstrated that the system created was more accurate than clinical cardiologists in classifying disease based on echocardiography alone, but further studies are needed to put the system into clinical application [86]. High-quality prospective evidence is still needed to show how the benefits of DL cardiovascular imaging systems can outweigh the risks [242].

#### 2.2.5. AI in Congenital Heart Disease

Congenital heart disease (CHD) is a field of application for AI, taking into account the diverse and robust datasets that extend from the management and diagnosis of pathologies to multimodal imaging. It has also increased the cohabitation of patients with CHD due to innovative surgery and new therapies. Thus, the use of AI could improve the quality of patient care, help optimize the treatment of these patients, extend life expectancy, save time for the attending physician, and reduce healthcare costs [243]. Artificial intelligence has found usefulness in current studies in the prediction of cardiovascular events in adults operated on for Fallot tetralogy [89] and the screening of congenital diseases based on ECG [90] based on cardiac auscultation [91] or echocardiography [93,94]. De Vries R.I. et al. conducted a study that developed an ECG-based fetal screening method for CHD. They demonstrated a 63% detection rate for all CHD types and 75% for critical CHD [90]. A study of 386 patients identified predictors of impaired executive function in adolescents after surgical repair of critical congenital heart disease (CHD), which were as follow: social class as the primary predictor and birth weight, neurological events, and number of procedures as other predictors [92]. 

#### 2.2.6. AI in Electrocardiography

Cardiovascular disease (CVD) crosses geographic, gender, or socioeconomic boundaries. Electrocardiograms (ECGs) are a routine instrument for any complete medical evaluation. ECGs are also used for diagnosis [244].

Artificial intelligence has found its usefulness in ECG for the following purposes: diagnosis of pulmonary thromboembolism [95], prediction of sudden death and cardiovascular events [97,98], prediction of fatal events after cardiac resynchronization [99], prediction of paroxysmal atrial fibrillation [37], detection of ventricular hypertrophy [100], risk prediction in liver transplantation [101], detection of ventricular dysfunction [103], and prediction of recurrence after paroxysmal atrial fibrillation ablation [38].

Valente Silva, B. et al. published a study on a batch of 1014 ECGs from patients presenting to the emergency room with suspected pulmonary embolism (PE). The authors demonstrated and validated a high-specificity pulmonary embolism prediction model for PE diagnosis based on artificial intelligence and ECG [95]. Shiraishi Y. conducted a study enrolling 2559 patients hospitalized for decompensated heart failure. Together with the authors of this study, they demonstrated the prediction of death in cardiac subjects using AI-ECG [97]. Zaver B. H. et al. published a retrospective study in 2023 that enrolled patients from a single center who were evaluated for liver transplantation or who underwent liver transplantation between 2017–2019. During this period, 3202 ECGs were available in the system, of which 1534 were available pre-transplant, 383 on the day of transplant, and 1284 post-transplant. A total of 719 ECGs from a total of 300 patients were analyzed, of which 533 were pre-transplant ECGs and 196 post-transplant ECGs. The study demonstrated AI-ECG performance in patients who were proposed for evaluation for liver transplantation, in addition, it demonstrated performance similar to that in the general population, but which was lower in the presence of an elongated QTc. ECG analysis using AI showed its usefulness in predicting post-transplant de novo AF. AI-based ECG assessment has also shown utility in predicting decreased left ventricular ejection fraction post-transplant. Therefore, AI-ECG can be a useful tool for patients proposed for liver transplantation, as a positive screening for a decreased SV ejection fraction or atrial fibrillation can raise alarm signals for the development of new post-transplant AF or cardiac dysfunction. Therefore, the importance of using a large dataset and artificial intelligence (AI) has increased significantly in medicine [101].

#### 2.2.7. AI in Heart Failure

Heart failure (HF) is increasing in prevalence along with the complexity of its diagnosis and treatment. The management and diagnosis of patients with HF require a huge amount of clinical information, leading to the accumulation of large amounts of data. However, traditional analytical methods are not sufficient to manage large datasets. From HF prediction to HF diagnosis, classification, prevention and management, AI has proven its usefulness [245].

Artificial intelligence has found its place in the prediction of heart failure in asymptomatic patients [106], in the diagnosis and treatment of patients with heart failure with reduced ejection fraction [110], in the diagnosis and treatment of patients with heart failure with low ejection fraction, in the detection of heart failure with preserved ejection fraction [111], and in the prediction of congestive heart failure [114].

Heart failure (HF) with preserved ejection fraction (HFpEF) is common and is associated with a high burden of mortality, morbidity, and high healthcare costs. Currently, compared with low-ejection-fraction HF (HFrEF), few medical therapies have been shown to improve cardiovascular outcomes in studies in patients with HFpEF. A study published by Segar M. et al. on 1767 patients has shown that cl analysis based on machine learning can identify the fenogroups of HFpEF patients with different clinical characteristics and also predict long-term results [107].

Almujalys. N discussed acute heart failure (AHF) monitoring in a study published in 2023. Together with all the study authors, they designed a remote health monitoring system to effectively monitor patients with AHF. This tool also helps both patients and doctors. It concerns Internet of Things (IoT) technology, which has revolutionized data colloquialization and communication by incorporating intelligent sensors that collect data from various sources. In addition, it uses artificial intelligence (AI) approaches to control a huge amount of data, which leads to better storage, management, use, and decision making. The created system monitors the clothing activities of patients, which helps to inform patients about their health status [105]. 

Kamio T. et al. published a study of 1416 patients who were admitted to the intensive care unit (ICU) for acute heart failure (AHF) and who received furosemide treatment. Using AI, they created a model that predicted in-hospital mortality and mechanical ventilation in patients hospitalized for AHF [116].

#### 2.2.8. AI in Heart Transplant

Regarding heart transplantation, artificial intelligence shows its usefulness in the following situations: in the prediction of post-heart-transplant events [118,128,129], the prediction of rejection after heart transplantation [118,119,122], the prediction of COVID-19 in heart transplantation [121] and pediatric heart transplantation [126], and the prediction of post-transplant survival [127].

One study claims that for patients with end-stage heart failure, heart transplantation remains the only chance of life. Medicine has come a long way, and the number of heart transplants has increased exponentially worldwide, but the number of heart donors is not big enough to meet the high demand. This brings up a particular issue of resource allocation. Artificial intelligence comes to the rescue and allows doctors to quantify the risk of rejection, accurately predict post-transplant prognosis, and determine waiting list mortality [117]. Briasoulis A. et al. published a study on a group of 18,625 patients (mean age 53 ± 13 years, majority male—73%), in which they analyzed the prediction of outcomes after heart transplantation. They concluded that 1 year after heart transplantation, there were 2334 (12.5%) deaths. Additionally, using AI, they demonstrated an ML-based model that proved its effectiveness in predicting post-transplant survival as well as acute rejection after heart transplantation [118]. The prediction of post-heart transplant rejection was also analyzed by Seraphin T.P. et al. in a study published in 2023, which included 1079 histopathology reports of 325 transplant patients in three centers in Germany. The authors detected patterns of cell transplant rejection in routine pathology, even when trained in small cohorts [119]. Since 2021, the rejection of cardiac alograft has been a serious concern in transplant medicine. It is well known that endomyocardial biopsy with histological examination is the gold standard in the diagnosis of rejection, but poor inter-pathology agreement creates important clinical uncertainty. Peyster E.G. et al. published a study that looked at 2472 endomyocardial biopsies, which concluded that the degrees of cellular rejection generated by histological analysis using AI are the same as those provided by expert pathologies [124]. 

In 2022, Ozcan I. et al. published a study in a cohort of 540 patients in which they looked at the patient’s physiological age based on ECG and correlated this information with the risk of post-heart transplantation mortality.

They were able to demonstrate that age-related cardiac aging after transplantation is associated with a higher risk of major cardiovascular events (MACEs), such as mortality, re-transplantation, and hospitalization for heart failure or coronary revascularization. The usefulness of this discovery is that the change in the physiological age of the heart could be an important factor in the risk of post-heart transplant MACE [120]. This study was reinforced by Morales J. R.’s study, which suggested that there may be an association with ECG cardiac age and one-year post-transplant events [128].

#### 2.2.9. AI in Hypertension

Artificial intelligence is increasingly being used in treating hypertension. In the highly scientific literature, numerous machine learning techniques are used to diagnose and detect numerous diseases: hypertension prevention [138], hypertension prediction [132,137,140,142], hypertension prediction in young patients [133], hypertension diagnosis [131,136], hypertension management and treatment [134,139,141], and hypertension variability [135]. Hypertension is found in 1.28 billion adults according to the World Health Organization (WHO). Hypertension has been found in adults aged 30 to 79 worldwide. Of adults with hypertension, about 42% are treatable. WHO data claim that about one in five adults worldwide has achieved optimal blood pressure control through treatment. Hypertension is also the leading cause of death worldwide [246].

Lopez-Martinez F. et al. performed a study that included 24,434 people aged over 20 years in the USA; they developed a neural network model in which they evaluated several factors and their relationship with the prevalence of hypertension. This study focused on using ANN to estimate the association between smoking, sex, age, BMI race, diabetes, and kidney disease in hypertensive patients. The results of this study show a specificity of 87% and sensitivity of 40%, with a precision of 57.8% and a measured AUC of 0.77 (95% CI [75.01–79.01]). The advantage of this study is that the results are more efficient than a previous study by other authors using another statistical model with similar characteristics that showed a lower calculated AUC than the present study (0.73). This model needs validation in other clinical settings, and further studies should include socio-demographic information to increase accuracy and integrate this model with clinical diagnosis [132].

Masked hypertension (MHPT) is ambulatory blood pressure that is not normal but exhibits instant normal blood pressure. Therefore, patients with MHPT are difficult to identify, and they remain untreated. Soh, D. C. K et al. developed a paper in which they analyzed a computational intelligence tool that used electrocardiogram (ECG) signals to detect MHPT. EI demonstrated that the best accuracy for the diagnosis of arthritic hypertension in the ECG signals was KNN, 97.70% [131].

Risk stratification remains an important step in hypertensive patients, especially if they are young patients. Wu X. et al. performed a study on a group of 508 patients, who were followed for an average period of 33 months. Two new ML techniques (RFE and XGBoost) were applied in the study to analyze the future risk of young patients diagnosed with hypertension. Baseline clinical data were analyzed, as well as a composite endpoint including all-cause death, coronary artery revascularization, peripheral artery revascularization, acute myocardial infarction, new-onset stroke, new-onset atrial fibrillation/atrial flutter, new-onset heart failure, sustained ventricular tachycardia/ventricular fibrillation, and end-stage renal disease. These patients were treated in a tertiary hospital. The performance of these models was then compared with that of a traditional statistical model (Cox regression model) and a clinically available model (FRS model). The study showed that the prognostic efficacy of the analyzed ML method was comparable to that of the Cox regression model; moreover, the efficacy of the analyzed ML method was higher than that of the recalibrated FRS model [133].

Herzog L. et al. studied a cohort of 16,917 participants, predicting antihypertensive therapeutic success with the help of AI. With an accuracy of 51.7%, the custom model developed by the authors was based on deep neural networks. The most successful treatment was a combination of an angiotensin-converting enzyme inhibitor and a thiazide (with 44.4% percent), and the angiotensin-converting enzyme inhibitor used alone was the most commonly used treatment (with 39.1%). These results may help with personalized treatment and better management of this pathology [141].

#### 2.2.10. AI in Pulmonary Hypertension

In the last four decades, a considerable number of registries have been published for pulmonary hypertension, which is a rare condition. These data have enabled the management and understanding of this pathology to be improved. However, to increase the understanding of the pathophysiology of pulmonary hypertension, prognostic scales are needed, as well as scales for verifying the transferability of the results from clinical trials in clinical practice. Although there are a huge amount of data from numerous sources, they are not always taken into account by registries. This is why machine learning (ML) provides a great opportunity to manage all these data and subsequently access tools that could help to make an early diagnosis. All of this functions to advance personalized medicine, especially the prognosis of the patient [247].

Many studies have focused on the effects of AI on pulmonary hypertension, from the prediction of this rare pathology in adults [147,150,155,161,162] or children [151,163,165] to the prediction of survival [154,167] or risk in patients with pulmonary hypertension [156], diagnosis of pulmonary hypertension [149,152,153,156,157,158,160,169], and the treatment of this disease [148].

In 2023, Griffiths M. et al. published a study of 1232 patients in *Circulation*, using data from multicentric registries. The authors developed a predictive model for pulmonary hypertension in children and also with the help of this model discovered a high-risk model for the time of intervention in these children. In the test cohort, the developed model showed very good results, with an AUROC of 87%, sensitivity of 85%, and specificity of 77% [165]. Another study used echocardiographic data to diagnose pulmonary hypertension (PH) in the pediatric population in a cohort of 270 newborns. The results of the study showed an average F1 score of 0.84 for predicting the severity of pulmonary hypertension in newborns, 0.92 for binary detection using a 10-fold cross-validation, 0.63 for predicting severity, and 0.78 for binary detection on the device held by the tests. The authors conclude that the learned model focuses on clinically relevant cardiac structures, motivating its use in clinical practice; at the time, this paper was the first to show automated pH assessment in newborns using echocardiograms [163]. 

In 2024, Anand V. et al. studied a cohort of 7853 patients who underwent cardiac catheterization and echocardiography and created an ML model for predicting PH using data from echocardiography.

The cohort age was 64 ± 14 years, of which 3467 (44%) were women and 81% (6323/7853) had a diagnosis of PH. The final trained model included 19 measurements and features from the echocardiogram. The model showed high discrimination for diagnosing PH (area under the characteristic operating curve of the receiver, 0.83; 95% CI, 0.80 to 0.85) in the test data. The accuracy, sensitivity, positive predictive value, and negative predictive values of the model were 82% (1267/1554), 88% (1098/1242), 89% (1098/1241), and 54% (169/313), respectively. The authors concluded that the PH could be predicted based on echocardiographic and clinical variables without using the regurgitation rate at tricuspid. Thus, machine learning methods seem to be promising for diagnosing patients with a low pH probability [169].

#### 2.2.11. AI in Infective Endocarditis

Infective endocarditis (IE) is a serious infectious disease that has high morbidity and mortality rates and severe complications. Severe complications include cardiac arrhythmias, embolic events, and valve ruptures leading to acute heart failure. Early risk assessment of patients with IE is crucial to optimize treatment. The prognosis of IE is influenced by many factors, including laboratory tests, clinical factors, cardiovascular and systemic imaging, and a combination of these. In addition, electrocardiographic changes may indicate advanced disease and thus predict high morbidity and mortality [15]. A dynamically modulated heart rate is considered to be a surrogate of the interaction between the parasympathetic and sympathetic nervous systems. This is measured by the variability or fluctuation in the time intervals between normal heartbeats (heart rate variability [HRV]). Inflammation is reflexively inhibited by the vagus, through activation of the hypothalamic–pituitary–adrenal axis, which causes cortisol secretion. Inflammation is also inhibited by the vagus and by vagus-sympathetic innervation of the spleen, where proinflammatory cytokines are no longer released from macrophages, which have in turn been signaled by single T cells. To highlight this mechanism, in 2023, Perek S. et al. published a study on a group of 75 patients with a mean age of 60.3 years from a tertiary center with a diagnosis of infective endocarditis. With the help of logistic regression (LR), it was determined whether laboratory, clinical, and HRV parameters were predictive of severe short-term complications (metastatic infection, cardiac injury, and death) or specific clinical features (staphylococcal infection and type of valve). The authors demonstrated that the standard deviation of normal heartbeat intervals (SDNN) and in particular the root mean square of successive differences (RMSSD), which were derived from very short ECG records, can be used for the prognosis of patients with IE [248].

In 2024, Christopher Koon-Chi Lai et al. discovered a new risk score comparable to existing scores but which is superior to clinical judgment; it applies to patients with *S. aureus* bacteremia (SAB). The authors looked at 15,741 patients with infective endocarditis, 658 of whom had a diagnosis of endocarditis-infective Staphylococcus aureus (SA-IE). The AUCROC was 0.74 (95% CI 0.70–0.76), with a negative predictive value of 0.980 (95% CI 0.977–0.983). Of all the features analyzed, four were the most discriminatory: history of infectious endocarditis, age, community onset, and valvular heart disease [170].

Another study by Galizzi Fae, I. et al. concluded that the most feared complications of infectious endocarditis are cardiovascular and neurological, and they are independently associated with high mortality. In addition to these complications, variables such as older age and elevated CRP levels are also associated with increased mortality. With the help of AI, it has been shown that intra-hospital mortality is determined by cardiovascular complications. Therefore, rapid identification of patients at high risk can prompt more aggressive treatment, which may decrease the mortality rate [172].

#### 2.2.12. AI in Ischemic Heart Disease

When it comes to ischemic heart disease, artificial intelligence can be useful both in the early diagnosis of ischemic heart disease [175,176,177,178,184,185] and in the prediction of complications after an acute ischemic event [186,187,188,189,190] and coronary artery disease [179,180,181,191]; AI can also be used in coronary artery disease prevention [11]. 

The current guidelines state that natural CAD can be modified by medical therapies, risk stratification, and early detection of CAD. In this way Ciccarelli M. et al. published an article in 2023 in which they mentioned (1) various machine learning algorithms based on single-photon emission computed tomography (SPECT) to facilitate CAD prediction and (2) prediction of major adverse cardiac events (MACE) in patients with the current or prior acute coronary syndrome (ACS) by risk scores such as the SINTAX87 score; however, these tools do not have the expected accuracy. The authors recalled in their study the use of machine learning techniques in identifying patients with increased morbidity and mortality following ACS. To estimate the risk of myocardial infarction, major bleeding, and, death of any cause for a period of 1 year, the PRAISE95 score was used and demonstrated precise discriminatory capabilities [11].

In patients with acute myocardial infarction (AMI), the most common cause of in-hospital death, despite early revascularization, is cardiogenic shock (CS) cauing 5–10% of deaths. Of all cases of CS, about 70% may be due to AMI. Bai Z. et al. published a paper in which five machine learning methods were analyzed to predict in-hospital cardiogenic shock in STEMI patients. These models include least absolute shrinkage and selection operator (LASSO), logistic regression (LR) models, support vector regression (SVM), and the tree-based ensemble machine learning models gradient boosting machine (LightGBM), and extreme gradient boosting (XGBoost). Of all the learning methods, the most successful prediction performance was represented by the LASSO model. The LASSO model in STEMI patients could provide excellent prognostic prediction for the risk of developing CS. The study included a group of 2282 patients with STEMI. The best overall predictive power was shown by linear models constructed using LASSO and LR, with an average accuracy of over 0.93 and an AUC of over 0.82. However, the LASSO nomogram showed adequate calibration and better differentiation, with a C-index of 0.811 [95% confidence interval (CI): 0.769–0.853]. A high C-index value of 0.821 was obtained for the internal validation tests. In terms of the decision curve (DCA) and clinical impact curve (CIC), the LASSO model showed superior clinical relevance compared to previous models that were score-based [41].

In order not to delay acute myocardial infarction (AMI) diagnosis, Liu W. C. et al. published a paper in which they developed a deep learning model (DLM) that analyzed 450 12-lead electrocardiograms (ECGs) for improved diagnosis of AMI. For STEMI detection, in the human–machine comparison, the AUC for DLM was 0.976. This was better than that of the best doctors. DLM also showed sufficient diagnostic capacity for STEMI diagnostics (AUC = 0.997; sensitivity, 98.4%; specificity, 96.9%) independently. Compared to NSTEMI diagnostics, the combined AUC of conventional cardiac troponin I (cTnI) and DLM increased to 0.978, which was superior to that of cTnI (0.950) or DLM (0.877). The authors concluded in their study that DLM can be used as a tool to help clinicians make an objective, timely, and accurate diagnosis for subsequent rapid initiation of reperfusion therapy [178]. 

Zhao Y. et al. also published a paper in which artificial intelligence (AI) proved able to provide a way to increase the efficiency and accuracy of ECG in STEMI diagnosis. They created an AI-based STEMI self-diagnostic algorithm that used a set of 667 ECG STEMI and 7571 control ECGs. The algorithm proposed in their study reached an area under the receiver operating curve (AUC) of 0.9954 (95% CI, 0.9885 to 1) with sensitivity (recall), specificity, accuracy, and F1 scores of 96.75%, 99.20%, 99.01%, 90.86% and 0.9372, respectively, in the external evaluation. In a comparative test with cardiologists, the algorithm had an AUC of 0.9740 (95% CI, 0.9419 to 1) and sensitivity (recall), specificity, accuracy, and F1 score values of 90%, 98% and 94%, 97.82% and 0.9375, respectively. Meanwhile, cardiologists had sensitivity (recall), specificity, accuracy, and F1 score values of 71.73%, 89.33%, 80.53%, 87.05%, and 0.8817, respectively [176]. Cho Y. et al. also published a paper in which they concluded that myocardial infarction (MI) could be detected quickly using electrocardiography (ECG) with 6 derivatives, not only ECG with 12 derivatives. The authors developed and validated an algorithm based on deep learning (DLA) for MI diagnostics. The EI analyzed a batch of 412,461 ECGs to create a variational autoencoder (VAE) that reconstructed the precordial ECGs with 6 derivatives [177].

Alkhamis M.A. et al. published a study that developed predictive models for adverse events in the hospital and at 30 days in patients with acute coronary syndrome (ACS). The authors analyzed 1976 patients with ACS and used clinical features and an interpretable multi-algorithm machine learning (ML) approach to match predictive models. EI demonstrated that the RF amplification algorithms and the extreme gradient (XGB) far exceed the traditional logistic regression model (LR) (ASCs = 0.84 and 0.79 for RF and RF, respectively, XGB). The most important predictor of hospital events was the left ventricle ejection fraction. From the point of view of events at 30 days, the most important predictor was the performance of an urgent coronary bypass graft. ML models developed by the authors of this study have elucidated nonlinear relationships that shape the clinical epidemiology of ACS adverse events and have highlighted their risk in individual patients based on their unique characteristics [187]. 

Kasim. et al. published a paper on 7031 patients in which they developed an ML model that improves mortality prediction accuracy by identifying unique characteristics within individual Asian populations. The performance of the algorithm created by the authors reached an AUC between 0.73 and 0.89. The TIMI risk score was exceeded by the ML algorithm, with superior performance for hospital predictions at 30 days and 1 year (with AUC values of 0.88, 0.88, and 0.81, respectively, all *p* < 0.001), while TIMI scores were much lower, at 0.55, 0.54 and 0.61. This finding shows that the TIMI score seems to underestimate the risk of mortality in patients. Key features identified for both short- and long-term mortality included heart rate, Killip class, age, and low-molecular-weight Heparin (LMWH) administration [189].

#### 2.2.13. AI in Pericardial Disease

AI has proven its usefulness in pericardial diseases; from the diagnosis of liquid pericarditis based on ECG [193] to the measurement of pericardial fluid based on echocardiography [194], automatic detection and classification of pericarditis using CT images of the chest [195], and prediction of fluid pericarditis in patients undergoing cardiac stimulation [196] or in breast cancer patients [192]. 

Liu Y.L. et al. published a retrospective study, being the first DLM study using a 12-lead electrocardiogram to diagnose acute pericarditis. The strategy developed by the authors is based on discriminating ECGs from acute pericarditis versus ECGs from STEMI in patients presenting with anterior chest pain to the emergency room. This study can be used as a basis for other larger studies and can also be an important support tool for the detection of pericarditis in the on-call room. This method can also be applied remotely and in telemedicine, as well as for portable technologies [193].

Piccini, J. P. et al. published a paper in which they determined predictive factors in which they developed a risk score for pericardial effusion in patients undergoing attempted Micra leadless pacemaker implantation. The authors analyzed a group of 2817 patients and concluded that the overall rate of pericardial effusion following Micra implantation is 1.1%. Using lasso logistic regression, the study authors developed a valid risk score for pericardial effusion composed of 18 preprocedural clinical variables. Using bootstrap resampling, future predictive performance and internal validation were estimated. External validation also benefited the scoring system, using data from the Micra Acute Performance European and Middle East (MAP EMEA) registry. There were 32 patients with pericardial effusion in the study [1.1%, 95% confidence interval (CI) 0.8–1.6%]. The authors demonstrated in the study that the rate of pericardial effusion increased with Micra implantation attempts in patients at medium risk *(p* = 0.034) but also in those at high risk *(p* < 0.001). After the Micra implantation attempt, the risk of developing pericardial effusion can be predicted with reasonable discrimination using preprocedural clinical data [196].

#### 2.2.14. AI in Peripheral Arterial Disease

Patients with a diagnosis of peripheral artery disease (PAD) have a high risk of metabolic events as well as cardiac events but are also at high risk of overall death. To improve outcomes in patients diagnosed with PAD, it is necessary to identify the disease early with prompt initiation of correct risk-managing treatment. McBanell R.D. et al. published a paper in JAHA in which they uncovered an AI algorithm that evaluated the posterior tibial arterial Doppler signal in patients with PAD, with the help of which they determined the patients with the highest risk of death from all causes, MALE, and MACE. A total of 11,384 patients were included in the study, out of which 10,437 underwent ankle–brachial index testing (medium age, 65.8 ± 14.8 years old, 40.6% women). Some 2084 of the patients were followed for 5 years, during which 447 of the patients died, 161 suffered MALE, and 585 suffered MACE events. Adjustments were then made for sex, age and Charlson comorbidity index, and the AI analysis of the posterior tibial artery waveform provided an independent prediction of mortality (hazard ratio [HR], 2.44 [95% CI, 1.78–3.34]), major adverse cardiac events (HR, 1.97 [95% CI, 1.49–2.61]), and major adverse limb events (HR, 11.03 [95% CI, 5.43–22.39]) at 5 years. Their analyses assisted clinicians in detecting peripheral arterial disease (PAD), which can lead to early modification of risk factors and their tailoring to each patient [197].

McBane II R.D. also published a study in which he addressed all major adverse cardiac events (MACEs) and limb events (MALEs), but compared to the previous study, the authors relied only on patients suffering from diabetes mellitus (DM). The authors of this study published in the Journal of Vascular Surgery are developing a tool that can diagnose PAD and predict clinical utility. Like McBanell R.D’s study, Doppler arterial waveforms were analyzed to diagnose PAD, but in this study, only patients with a diagnosis of DM were analyzed. This study aimed to identify patients with diabetes who are at highest risk of PAD. Of the 11,384 patients analyzed, only 4211 patients with DM met the study entry criteria (mean age, 68.6 ± 11.9 years; 32.0% female). In the validation set, there was a final subset of testing that included 856 patients. Over 5 years, there were 319 MACEs, 99 MALEs, and 262 patients who died. An independent prediction of death was provided by patients in the upper quartile of prediction based on deep neural network analysis of the posterior tibial artery waveform (hazard ratio [HR], 3.58; 95% confidence interval [CI], 2.31–5.56), MACE (HR, 2.06; 95% CI, 1.49–2.91), and MALE (HR, 13.50; 95% CI, 5.83–31.27).

The authors also concluded that an AI analysis of the arterial Doppler waveform allows the identification of major adverse outcomes, MACEs, and MALEs (including all-cause death) in patients with DM [202].

Masoumi Shahrbabak et al. published a similar paper in which they investigated the feasibility of diagnosing peripheral artery disease (PAD) based on the analysis of non-invasive arterial pulse waveforms. We generated realistic synthetic blood pressure (BP) and pulse volume recording (PVR) waveform signals related to PAD present in the abdominal aorta with a wide range of severity levels using a mathematical model simulating arterial circulation and arterial BP-PVR relationships. We developed a deep learning (DL)-compatible algorithm that can diagnose PAD by analyzing brachial and tibial PVR waveforms and evaluated its effectiveness compared to the same DL-compatible algorithm based on brachial and tibial arterial BP waveforms and the ankle–brachial index (ABI). The results suggested that it is possible to detect PAD based on DL-triggered PVR waveform analysis with adequate accuracy, and its detection efficacy is close to that using blood pressure (positive and negative predictive values in 40% abdominal aortic occlusion: 0.78 vs. 0.89 and 0.85 vs. 0.94; area under the ROC curve (AUC): 0.90 vs. 0.97). The authors concluded that in the diagnosis of PAD, non-invasive arterial pulse wave analysis can be used with the help of DL as it is a non-invasive and accessible means [201]. 

#### 2.2.15. AI in Thromboembolic Disease

In thromboembolic disease, artificial intelligence has a role, especially in disease prediction [205,206,207,209,210,211,212,213]. AI is also used in the diagnosis of pulmonary embolism [95] and the diagnosis of deep vein thrombosis [214,215].

Valente Silva B. et al. published a paper in 2023 in which they developed and validated a 12-lead ECG-based deep learning model for the diagnosis of pulmonary embolism. This model shows a high specificity guard in the diagnosis of pulmonary embolism. The authors of the study looked at 1014 ECGs from patients who underwent pulmonary angiography due to suspected pulmonary embolism. Of all these patients, 911 ECGs were used to develop the AI model, and 103 ECGs were used to validate the model. The performance of the AI model used by the authors in this study was compared with the clinical prediction rules recommended by the guidelines in place for EP, such as the Wells and Geneva scores combined with a standard D-dimer threshold of 500 ng/mL and an age-adjusted threshold. The authors concluded that the AI model they developed reached a much higher specificity for diagnosing PE than the commonly used clinical prediction rules. So, the AI model showed 100% specificity (95% confidence interval (CI): 94–100) and 50% sensitivity (IC of 95%: 33–67). Compared to the other models, which had no discriminatory power, the AI model worked much better (area under the curve: 0.75; IC 95% 0.66–0.82; *p* < 0.001). In patients with and without PE, the incidence of typical PE ECG characteristics was similar [95].

Seo J W et al. also addressed the diagnosis of deep vein thrombosis using AI methods and performed a study in which they evaluated the performance of an artificial intelligence algorithm (AI) for the diagnosis of iliofemoral deep vein thrombosis. They used computed tomographic angiography of the lower extremities. The authors concluded that the profuse is an effective method of reporting critical phases of iliofemoral deep vein thrombosis [215]. Contreras-Lujan, E. E. et al. supported previous and public research and used ML methods for more reliable and efficient DVT diagnosis to be incorporated into a high-performance system to develop an intelligent system for the early diagnosis of DVT. The authors concluded that the accuracy of all models trained on PC and Raspberry Pi 4 was greater than 85%, while the area under the curve (AUC) was between 0.81 and 0.86. So, for diagnosing and predicting early DVT, ML models are effective compared to traditional methods [214].

Nassour N. et al. also published a paper in 2024 in which they evaluated new automatic learning techniques to estimate the risk of VTE and the use of prophylaxis after ankle fracture. The authors analyzed using machine learning and conventional statistics 16,421 patients who suffered ankle fractures and were evaluated retrospectively for symptomatic VTE. Of all the patients, 238 patients with VTE confirmed later in the 180 days after the injury either sustained conservative or surgical treatment for ankle fracture. In the control group, there were 937 patients who had no evidence of VTE but who had ankle fractures and had similar treatment. Patients in both groups were divided into those receiving VTE prophylaxis and patients not receiving VTE prophylaxis. More than 110 variables were included. The results of the study were that the higher incidence of VTE was in the group of patients who underwent surgical treatment for ankle fracture, those who had increased hospitalization, and those who were treated with warfarin. The authors concluded that when machine learning was applied to patients with ankle fractures, several predictive factors were successfully found to be related to the appearance or absence of VTE [205].

#### 2.2.16. AI in Valvular Disease

Artificial intelligence seems to be promising in valvular diseases; in this review, we focused our attention mainly on aortic diseases [216,217,222,223,224,225,228] and aortic dissection [226,227] as well as aortic aneurysm [229] and rheumatic diseases, focusing on mitral regurgitation [220]. 

For the treatment of aortic stenosis, transcatheter aortic valve replacement (TAVR) is the procedure increasingly used. Toggweiler, S. et al. have developed automated software to make the necessary measurements for planning TAVR with high reliability and without human help. The authors compared the automatic measurements from 100 CT images with the images from three TAVR expert clinicians. It was noted that the aortic ring measurements generated by AI had very good agreements with those performed manually by doctors, with correlation coefficients of 0.97 for both the perimeter and the area. For the measurement of the ascending aorta at 5 cm above the ring plane, the average difference was 1.4 mm, and the correlation coefficient was 0.95 [221]. 

Xie, L.-F. et al. published a study in 2024 integrating artificial intelligence to build a predictive model of postoperative adverse events (PAOs) based on clinical data. They wanted to evaluate the incidence of PAO in patients operated with acute aortic dissection type A (AAAD) after total arch repair. The authors included a group of 380 patients with AAAD in the study. They used LASSO regression analysis. After a thorough analysis, the authors concluded that the most optimal model is the extreme gradient growth model (XGBoost) as it showed better performance than other models. Therefore, for patients with AAAD, the prediction model for PAO is based on the XGBoost algorithm, and this model is also interpreted via the SHAP method. This method helps clinicians to identify high-risk AAAD patients at an early stage and choose optimal individualized treatment [226].

Brown, K. et al. published a paper in 2024 in JAHA concluding that artificial intelligence could detect rheumatic heart disease (RHD) in children as well as expert doctors. The authors included 511 ultrasounds from children in their studies, with color Doppler images of the mitral valve. Ultrasound scans were also evaluated by a group of expert doctors. RHD was present in 282 cases out of 511, and 229 were normal. The automatic learning method developed by the authors identified the correct vision of the mitral regurgitation jet and the left atrium, with an average accuracy of 0.99, and the correct systolic frame with an average accuracy of 0.94 (apical) and 0.93 (parallel long axis) [220].

## 3. Discussion

AI has broad application prospects in cardiovascular disease, and a growing number of scholars are devoted to AI-related research on cardiovascular disease. Cardiovascular imaging techniques (electrocardiography and echocardiography) and the selection of appropriate algorithms (ML or DL) represent the most extensively studied areas, and a considerable boost in these areas is predicted in the coming years. 

Strengths: Cardiology leads the way in the artificial intelligence revolution in medicine. AI enables precise prediction of cardiovascular outcomes, non-invasive diagnosis of coronary artery disease, and detection of malignant arrhythmias. Additionally, it facilitates the diagnosis, treatment, and prognosis of heart failure patients. Advances in artificial intelligence and precision medicine will drive future innovations in cardiovascular research.

Limitations: Ethical and data privacy concerns are significant limitations to the widespread adoption of artificial intelligence in cardiology and medicine, requiring careful consideration. Regulations are needed to ensure the safe use of artificial intelligence in cardiology and medicine in the future.

### 3.1. Perspectives and Directions for the Application of Artificial Intelligence in Cardiology

Artificial intelligence (AI) has been integrated into the healthcare industry as a new technology that uses advanced algorithms to synthesize necessary information from huge databases. Research in the field of AI on cardiology has grown exponentially, as can be seen from the number of articles reviewed above. Arrhythmias, ischemia, diseases of the heart valves, heart failure, myocardial infarction, and problems affecting the peripheral arteries and the aorta are all examples of cardiovascular diseases (CVDs) [249].

A significant number of papers have been published in the field of structural heart disease, especially in the field of cardiomyopathies and ischemic heart disease, but also in pulmonary hypertension. At the opposite end of the spectrum, with a relatively smaller number of articles, is research in the field of AI-based arrhythmia and infective endocarditis. Current research also focuses on machine learning, especially in the use of ECG signals and echocardiograms. As an indispensable tool in cardiology, ECG has become one of the most useful tools for collecting data as input for ML, just like echocardiography. In addition, the role of other instruments that collect data, such as coronary angiography, cardiac MRI, or cardiac CT, should not be minimized. Thus, cardiovascular imaging is one of the main sources of information which is far from being at full capacity. In addition, a tremendous amount of data can come from laboratory data, and hospitals can provide the researcher with data on both patient history and patient profile. These opportunities should be exploited closely, as there is great untapped potential at this time. 

Convolutional neural networks, recurrent neural networks, and cross-validation are types of AI much more widely used in publications relevant to this paper, as compared to other machine learning techniques. Deep learning is more widely used in general, compared to unsupervised ML or classical ML models. There are also papers in which predictive values are low, although the negative predictive values are high, which raises the issue of further refinement and further development of these systems. The authors of this article believe that in technology research, close collaboration between AI engineers and clinicians makes effective decision making possible. One potential area of future development is engineering in medical AI and medicine; there will probably be discussion in the future about physicians with exhaustive knowledge of medical AI. For significant technological progress and innovation, close collaborations between healthcare engineering systems and physicians are needed [250].

Finally, through this paper, we also wish to highlight some perspectives for future research, perhaps answering questions about legal and ethical considerations. Who decides whether an AI diagnostic system is safe for the patient, government hospitals, or individual hospitals? Who is directly responsible and who is investigated when a malpractice case is taken up: engineers, technology companies, doctors, or hospitals? What can be done about patients’ data privacy and who should be trained to protect it? How can we prevent doctors’ judgmental standards from falling due to reliance on AI for diagnosis, which may become a serious problem in a few generations [250]? For correct and complete implementation, this side of AI must be addressed, and for the moment, it is one of the most sensitive issues. Scientific knowledge in the field of artificial intelligence in cardiology is, as we have seen in the analysis carried out, in continuous ascension, and different methods are already being implemented all over the world. We can subdivide these methods into several essential aspects: (1) prevention of cardiovascular diseases; (2) screening; (3) diagnosis of cardiovascular diseases; and (4) treatment, all of which function for the adult population and the pediatric population. 

#### 3.1.1. Prevention

Preventive cardiology can be seen today as an understudied specialty within cardiovascular treatments. Preventive cardiology aims to improve the known risk factors for CV disease (CVD). Preventive cardiology has also found a use for AI [11], as AI can introduce new treatment methods and important tools to assist the cardiologist in reducing the risk of CVD. The role of AI has been investigated in weight loss, sleep, nutrition, physical activity, dyslipidemia, blood pressure [138], alcohol, smoking, mental health, and recreational drugs. AI has huge potential to be used for the detection, screening, and monitoring of the mentioned risk factors. However, in terms of preventive cardiology, there is a need for the literature to be complemented by future clinical trials addressing this issue [251].

In cardiovascular disease prevention, artificial intelligence has found its place in several areas; it has an important position in precision cardiovascular disease stratification, integration of multi-omics data, discovery of new therapeutic agents, expanding physician effectiveness and efficiency, remote diagnosis and monitoring, and optimal resource allocation in cardiovascular prevention. The newest applications of artificial intelligence in cardiovascular prevention are addressing the main cardiovascular risk factors, in particular dyslipidemia, hypertension, and diabetes [11]. 

Diabetes carries twice the risk of coronary heart disease, vascular death, and major stroke subtypes, which is why controlling risk factors, especially diabetes, is crucial from childhood. The age of onset of diabetes is steadily decreasing, with one study noting an age of onset of 6–12 years. The study authors also conclude that glycemic balance in children in particular is increasingly difficult to maintain. This study shows statistically significant differences (*p* < 0.05) in terms of mean systolic SBP values with type I diabetes and type II diabetes, which confirms the importance of controlling cardiovascular risk factors from childhood for the prevention of cardiovascular disease, especially as the study also recommends monitoring lipid profile from childhood and applying therapeutic measures [252].

Japanese researchers used a machine learning approach that looked at more than 18,000 patients, and they developed an algorithm with increased sensitivity for predicting new-onset hypertension that demonstrated greater accuracy than the usual logistic regression model, reaching an AUC close to 0.99 [253]. Another larger study confirmed previous results. It included more than 8,000,000 people from East Asia using an open-source platform with potential large-scale applicability [254]. 

In dyslipidemia, artificial intelligence has tested applications from diagnosis to the management and prognosis of dyslipidemia. Recent studies have demonstrated the possibility of cardiovascular risk assessment using deep learning, which helps to estimate LDL cholesterol with better accuracy using machine learning [255]. In addition, recent predictive methods for incidental dyslipidemia have been obtained by modeling machine learning on larger datasets considering monogenic or polygenic variants [256,257]. 

A similar study has pointed out that in screening programs, the use of triglycerides to estimate cardiovascular risk is also recommended from childhood. However, caution should be exercised, as elevated values may be falsely elevated, especially in women with high HDL or in patients with metabolic syndrome or diabetes where low HDL levels may occur frequently. Extrapolating from the above information, future studies may address the analysis of triglyceride values using AI to better control cardiovascular risk factors for optimal cardiovascular disease screening [258].

#### 3.1.2. Screening

A recent article has discussed screening for cardiovascular disease in women using AI [259], this being a subcategory analyzed by the authors within the wide range of areas in which AI has proven effective in screening (e.g., congenital disease screening from both ECG analysis [90] to heart sound analysis [91] and fetal ultrasound [93], screening for reduced fraction heart failure [109], screening for hypertension [138], screening for valvular disease [218,219,220,222,227,228], and screening for rare diseases such as Fabry disease [60]).

Even though the potential opportunities for AI in CVD screening are enormous, further research is needed to objectively assess whether digital technologies improve patient outcomes [260].

#### 3.1.3. Diagnosis 

When it comes to cardiovascular diseases, AI also plays an important role in their diagnosis. From the acute diagnosis of left ventricular hypertrophy using imaging methods such as echocardiography [56], to the diagnosis of amyloidosis also based on TTE [66] or idiopathic pulmonary hypertension [157], artificial intelligence has demonstrated its power to help clinicians.

**Figure 3:**
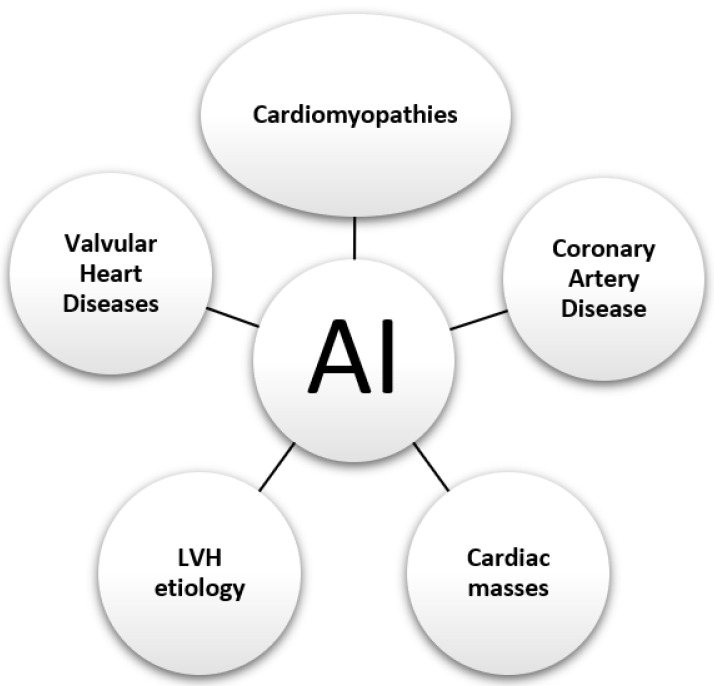
The usefulness of artificial intelligence in echocardiography for diagnosing disease. Created based on information from [261].

Echocardiography is an imaging method that detects certain abnormalities in real time and is also one of the few imaging methods that allows real-time imaging. Although artificial intelligence has been around since the 1950s, a major focus in recent years has been on the application of AI to diagnostic imaging. Machine learning and other AI techniques can drive a variety of patterns in imaging modalities, particularly echocardiography [261]. The potential clinical applications of AI in echocardiography have increased exponentially, including the identification of specific disease processes such as coronary heart disease, valvular heart disease, hypertrophic cardiomyopathy, cardiac masses, and cardiac amyloidosis and cardiomyopathies (Figure 3).

In the valvular heart disease subcategory, the focus of AI is on identifying high-risk patients and echocardiographic quantification of the severity of valvular disorders [262]. VHD refers to problems with mitral, aortic, pulmonary, or tricuspid valves. Treatment and identification of cardiovascular diseases could be significantly improved by the application of AI. AI has used various types of echocardiography, ECG, phonocardiography, and ECG to help diagnose valvular diseases. 

In this review, we focused our attention on aortic diseases and very little on the mitral valve. Assessment of aortic valve disease’s progression can be carried out using AI-based algorithms that integrate the data from the evaluation echocardiography of the aortic valve with additional clinical information [263]. Transcatheter valve replacement decisions, such as the right valve size and selection, can be improved by using AI to automate the measurement of anatomical dimensions derived from imaging data [221]. 

Recently, a study that included nearly 2000 patients diagnosed with aortic stenosis concluded that AI helped to identify high-risk patients and improved the classification of aortic stenosis severity by integrating echocardiographic measurements. Additionally, identifying subjects at higher risk in this study (patients who had high levels of biomarkers, higher calcium scores of the aortic valve, and higher incidence of negative clinical outcomes) could optimize the timing of aortic valve replacements [264].

Another study, including 1335 test patients and a validated cohort of 311 patients for validation, developed a tool for the automatic screening of echocardiographic videos for aortic and mitral disease. This deep learning algorithm was able to detect the presence of valvular diseases, classify echocardiographic opinions, and quantify the severity of the disease with high accuracy (AOC > 0.88 for all left heart valve diseases) [265]. All of these findings support the effectiveness of a tool to be trained on routine echocardiographic datasets to classify, quantify, and examine the severity of conditions most common in medical practice.

Furthermore, the potential of AI in developing algorithms for CVD diagnosis and prediction will receive major research attention in the coming years. Thus, the application of AI in the field of CVD has gained significant momentum, especially in the diagnosis of coronary heart disease but also in the classification of cardiac arrhythmias, which is a future trend. In addition to echocardiography, other non-invasive imaging techniques such as cardiovascular magnetic resonance imaging (CMRI) possess robust computing power, as well as large datasets and advanced models. In today’s world, this is the cornerstone of cardiovascular diagnostics. CMRI is a widely used and accepted tool for assessing cardiovascular risk. It incorporates AI, especially in image recognition and in revolutionizing cardiomyopathy prognostic analyses using late gadolinium enhancement (LGE) [266].

The role of AI extends to minimizing artifacts in CMRI and identifying scar tissues [73,173], thereby increasing diagnostic accuracy and speed [71,153,174]. Studies such as those using RF differentiate hypertrophic from dilated cardiomyopathies and also from healthy patients via CMR analysis [71].

Studies examining ischemic coronary artery disease [11,173,174,175,176,177,178,179,180,181,182,183,184,185,186,187] use AI both in predicting the disease [180,181,191] and in its diagnosis [179,183,184] or prognosis [41,186,187,188,189,190]. Other studies use machine learning models in patients undergoing coronary artery bypass graft (CABG) surgery to create predictive models of the risk of continuous renal replacement therapy (CRRT) after surgery [267].

A recent study has addressed the topic of CABG patients, who are often frail patients with multiple comorbidities, including chronic obstructive pulmonary disease (COPD), sleep apnea, high blood pressure, and diabetes. COPD is currently one of the most worrying and significant public health problems in many countries. COPD causes an estimated 3.5 million deaths annually and affects over 600 million people worldwide [268]. The most commonly implemented AI algorithms in the diagnosis, prevention, and classification of COPD disease are decision trees and neural networks [269]. 

In patients with diabetes mellitus, atherosclerotic coronary artery disease is even more common but often more advanced. In these cases, the benefits of percutaneous interventions, which may have a higher risk of in-stent restenosis, have been outweighed by CABG surgery. The authors’ perspective thus contributes to a nuanced view of post-CABG outcomes in these patients through appropriate drug treatments but also through post-CABG rehabilitation programs in patients included in their study with/without type 2 diabetes and with/without chronic kidney disease. They demonstrate the clear superior benefit of innovative treatment in cardiology, the SGLT2 inhibitor, which was used during a cardiovascular rehabilitation program and reduced ischemic risk in patients included in their study. This study may represent future research directions in the field of AI in cardiology in patients with ischemic heart disease, especially since the authors mention that their paper is the first in the literature to address this topic (the impact of SGLT2 inhibitors on CABG patients with/without chronic kidney disease and with/without type 2 diabetes mellitus who are undergoing a cardiac rehabilitation program) [270]. There are already studies that have relied on machine learning models that have been designed to perform virtual screening in terms of exploring sodium–glucose cotransporter (SGLT2) inhibitors using AI. The authors have already raised some future research topics, such as identifying new types of drugs as possible next-generation SGLT2 inhibitors and chemotherapy [271].

#### 3.1.4. Treatment 

As far as the treatment of cardiovascular disease is concerned, artificial intelligence has found its place even in acute treatment, such as in patients with cardiogenic shock treated with ECMO. When the authors analyzed a group of 258 elderly patients with cardiogenic shock, the mortality rate at 6 months after ECMO treatment was 52 patients (20.16%). Using algorithms, predictive models were constructed to determine the mortality rate and prognosis of the patients in the study. The accuracy, sensitivity, and specificity of the random forest (RF) model were 0.987, 1.000, and 0.929, respectively, which were higher than those of the decision tree model [49]. Additionally, in the treatment of chronic diseases via treatment paradigms for patients with heart failure with acute kidney disease, the authors outlined how AI technologies can be adapted to address major issues among HF patients with acute kidney injury. They identified both personalized interventions and treatment planning using AI without real-time monitoring. In addition, they drew attention to the need for validation and the importance of collaboration between cardiologists and nephrologists [115].

Artificial intelligence has also found its place in the treatment of hypertension [134] and in the treatment of COVID-19 in patients with pulmonary hypertension [148]. The authors discuss patients’ adherence to antihypertensive treatment and suggest through this paper that artificial intelligence is an effective alternative to conventional methods for understanding treatment adherence. This finding may be used as a useful tool in educating patients about the importance of medication in the management of hypertension [134].

Artificial intelligence is also involved in the treatment of patients with AMI [175] or acute aortic dissection [226]. The authors created a predictive model based on XGBoost that aims to identify high-risk AAAD patients and develop individualized treatment and diagnostic plans to improve the prognosis of patients diagnosed with AAAD.

To predict the future, we should probably visualize the potential limitations and shortcomings of artificial intelligence at the current stage, as these important elements will have the power to guide us toward new research that will lead to new advances in the years to come. As far as cardiac ultrasound is concerned, AI algorithms are based on datasets that already exist in the real world but which carry the same risks and limits of possible misclassification, the presence of arrhythmias (difficult to handle by artificial intelligence models), and the possibility of sub-optimal image quality (implying limited authenticity or exclusion of some acquisitions, and therefore limited authenticity) when detecting wall motion abnormalities. Additionally, given the frequently inadequate standardization datasets and the limited number and representativeness of datasets, automated software is currently inferior to semi-automatic software in terms of measuring anatomy and morphofunctional structure [272].

### 3.2. Ethical Considerations of AI in Cardiology

When it comes to artificial intelligence in cardiology, ethical concerns take center stage, especially regarding the privacy of patient data and algorithmic biases. The introduction of AI in cardiology prompts worries about how patient data, often large and sensitive, will be handled to train and test these algorithms. Protecting patient privacy is crucial to maintain trust in the healthcare system. Moreover, there is the issue of algorithmic biases, which can arise from the data used to train AI models. These biases could lead to disparities in healthcare, affecting everything from diagnosis to treatment outcomes. To tackle these ethical challenges, we need transparency in AI development, robust data protection measures, and ongoing efforts to detect and correct algorithmic biases. It is also vital for healthcare professionals, data scientists, ethicists, and policy makers to work together closely to ensure that AI in cardiology is used responsibly and fairly.

### 3.3. Bias Risk Assessment

A significant concern in the use of AI in cardiology is the risk of bias that can affect outcomes and interpretations. Bias can occur at several stages of the process, including data collection and selection, algorithm construction, and result interpretation. For instance, the input data used for training algorithms may be influenced by population characteristics, collection methods, or human errors. Additionally, the algorithms themselves can be affected by implicit biases embedded in the datasets or in the training process. This can lead to distorted results or incorrect generalizations, compromising the effectiveness and reliability of AI systems in diagnosing and treating cardiac conditions. Therefore, it is crucial to conduct a careful assessment of bias risk in studies utilizing artificial intelligence in cardiology and to apply appropriate methods to minimize and manage this risk.

For a robust design of cardiovascular disease prediction based on machine learning, it is crucial to consider the following aspects: (i) the use of stronger outcomes, such as death, calcium arterial coronary score, or coronary stenosis; (ii) ensuring scientific and clinical validation; (iii) adapting to multi-ethnic groups while practicing unseen AI; and (iv) amalgamating conventional, laboratory, imaging, and pharmacological biomarkers. In the studies we analyzed from the high-quality scientific literature, all these aspects have been assessed and accounted for.

Summary of findings from the papers reviewed:

Common themes: The integration of AI in cardiology has seen substantial growth, particularly in addressing various cardiovascular diseases (CVDs) such as arrhythmias, ischemia, and heart failure. Significant focus on structural heart disease, cardiomyopathies, and ischemic heart disease, alongside emerging areas like pulmonary hypertension, indicates diverse research interests. Utilization of machine learning techniques, especially in analyzing electrocardiogram (ECG) signals and echocardiograms, highlights the importance of AI in data analysis for diagnostic purposes. There is emphasis on the role of cardiovascular imaging techniques, including ECG, echocardiography, coronary angiography, and cardiac MRI, as essential sources of information for AI applications in cardiology.

Challenges: Despite advancements, some AI models exhibit low predictive values, showing the need for further refinement and development. Ethical and legal considerations regarding the safety of AI diagnostic systems, patient data privacy, and potential overreliance on AI for diagnosis pose significant challenges.

Areas of consensus: Collaborative efforts between AI engineers and clinicians are deemed essential for effective technological progress and innovation in medical AI. Future research directions emphasize preventive cardiology, screening, diagnosis, and treatment of cardiovascular diseases using AI, catering to both adult and pediatric populations.

## 4. Conclusions

The use of artificial intelligence (AI) in the field of cardiovascular diseases represents an emerging paradigm in modern medicine, offering significant advantages in the diagnosis, prognosis, and management of these conditions. From the early identification of thromboembolism and pericarditis to the comprehensive evaluation of valvular and ischemic diseases, AI algorithms provide an essential contribution to improving diagnostic efficiency and clinical decision making.

In the case of thromboembolic diseases, AI algorithms demonstrate an impressive capacity to predict the risk of thromboembolic events and assist in the precise diagnosis of pulmonary embolisms and deep vein thromboses. By identifying subtle patterns in electrocardiographic and medical imaging data, AI enables early detection and prompt intervention, significantly enhancing patient management.

Regarding valvular diseases, AI offers advanced tools for assessment and treatment planning, such as transcatheter aortic valve replacement (TAVR). AI algorithms can make precise and reliable measurements, comparable to those performed manually by physicians, optimizing the decision-making process and ensuring better outcomes for patients.

On the other hand, in pericardial diseases, AI facilitates diagnosis and prognosis, providing a faster and more accurate approach to evaluating ECGs and echocardiographic images. By identifying subtle signs and characteristic patterns, AI algorithms enable early identification of pericarditis and pericardial effusions, contributing to improving patient management.

Last but not least, artificial intelligence holds immense promise in revolutionizing the management of ischemic heart disease, offering enhanced diagnostic accuracy, risk prediction capabilities, and personalized treatment strategies. Its application in cardiovascular care signifies a paradigm shift towards more precise and tailored approaches, ultimately improving patient outcomes and optimizing healthcare delivery.

The use of deep learning algorithms and data processing techniques contributes to optimizing clinical decisions and improving outcomes for patients. However, rigorous implementation and validation are essential to ensure the safety and effectiveness of these technologies in clinical practice.

## Figures and Tables

**Table 1:** Important AI-related terms and definitions.

Term	Definitions	References
Artificial intelligence (AI)	Artificial intelligence (AI) is a subtype of information technology that through algorithms can analyze (receive, process, and interpret) medical information and perform complex mathematical calculations, simulating artificially what happens in the human mind during learning.	[14]
Machine learning (ML)	Machine learning (ML) is the ability of computer systems to automatically learn from existing data and past experiences to find patterns and make future predictions. ML is a well-known subtype of AI and can be grouped into three categories: supervised learning, unsupervised learning, and reinforcement learning. In medicine, ML can incorporate and manage various data resources (from clinical and biological observations to wearable devices and environmental information) to create models that can predict and diagnose certain diseases. Additionally, ML can personalize disease treatment to improve the healthcare system. In conclusion, ML is one of the fastest, most convenient, and cost-effective ways of detecting disease through artificial intelligence technology.	[15,16,17,18]
Deep learning (DL)	Deep learning (DL) is a subtype of machine learning that can analyze massive amounts of data to provide greater accuracy in creating concepts and accurately predicting pathologies. DL is currently one of the most applied algorithms for medical purposes, alongside support vector machine (SVM) and artificial neural network (ANN).	[4,19]
Cognitive computing	Cognitive computing systems are artificial intelligence systems that are part of machine learning and understand, reason, and enhance human brain capabilities by combining virtual technology and natural language processing.	[20]
Supervised learning	Training the ML algorithm using labeled examples consisting of inputs and outputs provided by an expert is a phenomenon known as supervised learning. Supervised learning encompasses artificial neural networks (ANNs), support vector machine (SVM), decision tree, random forest, fuzzy logic, naive Bayes (NB), K-nearest neighbor (KNN), and regression.	[17,21]
Unsupervised learning	This involves training the ML algorithm to process data and perform classification of samples without category information, thus without human intervention. Unsupervised learning includes clustering algorithms and association rule-learning algorithms.	[21,22]
Reinforcement learning	Reinforcement learning is a subtype of machine learning that can be considered a combination of supervised and unsupervised learning and can facilitate efforts to increase algorithm accuracy. It is a learning strategy for optimal learning regarding a specific criterion in a given situation. This algorithm receives feedback on its performance by comparing rewards obtained during training with the chosen criterion.	[23,24]
Convolutional neural networks (CNNs)	Deep learning (DL), a method primarily used in image processing and understanding or classifying images, involves models similar to those used in the visual cortex for processing images. Convolutional neural networks (CNNs) are neural networks similar to regular neural networks, as they are composed of neurons with weights that can be learned. However, CNNs explicitly assume that inputs have specific structures, such as images.	[21,25,26]
Recurrent neural networks (RNNs)	RNNs are different from CNNs in that the input data are of variable size, which can be processed by the RNN; moreover, the outputs of intermediate-layer neurons are cyclically captured in the original input. When many recurrent neurons exist in a recurrent layer, the sequential data are processed in parallel through different weights, allowing RNNs to generate multiple representations and create effective feature space separation.	[27]
Deep neural networks (DNNs)	A DL architecture with multiple layers between the input and output layers.	[21]
Artificial neural network (ANN)	An ML technique that processes information in an architecture comprising many layers (“neurons”), with each interneuronal connection extracting the desired parameters incrementally from the training data.	[21,28]
Support vector machine (SVM)	A supervised learning model that can efficiently perform linear and nonlinear classifications, implicitly mapping their inputs into high-dimensional feature spaces.	[29]
Decision tree (DT)	This nonparametric supervised learning method is visualized as a graph representing the choices and their outcomes in the form of a tree; each tree consists of branches (values that a node can take) and nodes (attributes in the group to be classified).	[30]
Random tree (RT)	This is an ensemble classification technique that uses “parallel ensembling”, fitting several decision tree classifiers in parallel on dataset subsamples.	[30]
Naïve Bayes (NB)	A classification technique assuming independence among predictors, Naive Bayes is a tool that works with the most basic knowledge of probability. Bayes’ rule is a formula that determines the probability that Y will happen with a given X. The Bayes technique makes the naive assumption of independence of all characteristics. It attempts to find probabilities based on known prior probabilities that have been learned from training data.	[29]
Fuzzy logic	Fuzzy logic is part of supervised learning which allows multiple possible truth values to be processed through the same variable.	[30]
K-nearest neighbor (KNN)	This non-generalizing learning algorithm or an “instance-based learning” does not focus on constructing a general internal model but rather stores all instances corresponding to the training data in an *n*-dimensional space and classifies new data points based on similarity measures.	[30]
Regression	This is an algorithm using a logistic function to estimate probabilities that can overfit high-dimensional datasets, being suitable for datasets that can be linearly separated.	[30]
Clustering algorithms	Data clustering is an essential part of extracting information from databases and is part of unsupervised learning. There are several ways to split the data, the most important of which are horizontal and vertical collaborative clustering.	[31]
Association rule-learning algorithms	Association rule learning and correlation learning methods are used to find and weigh contextual relations between modeled context entities. In the presence of a training dataset, a unique classification strategy is introduced, which can effectively increase classification performance.	[32,33,34]

**Table 2:** Scientific articles that analyze the current applications of AI in cardiology as well as future research perspectives.

	Year of Study	Author	Application	Data Source	Machine Learning Method	Future Direction
Arrhythmias	2023	Tran, K.-V. [35]	AF detection	mECG	CNN	Studies to improve the AI algorithm of commercial wearable devices for AF detection
	2023	Baj, G. [36]	Prediction of new-onset AF	ECG	CNN XGB LR	Integration of demographic information (gender, age) or clinical information as predictors in addition to ECG; clinical interpretability is a fundamental step to building predictive tools for clinical usage
	2023	Raghunath, A. [37]	Prediction of AF	mECG	CNN DNN	Differentiating atrial fibrillation from atrial flutter using the availability of information such as clinical indicators, socio-economic status, or racial background
	2023	Jiang, J. [38]	Prediction AF recurrence 12 months after catheter ablation	ECG	CNN	Larger batches of patients are analyzed to improve applicability and accuracy. In addition, a prospective study is needed; other studies assess recurrence after more than 12 months
	2021	Bai, Y. [39]	Prediction of AF recurrence after catheter ablation	ECG	CNN	To generalize the premise, larger batches of patients were analyzed, with more pathologies
Cardiogenic Shock	2022	Rahman, F. [40]	Predicting patients at high risk of developing cardiogenic shock (CS)	Demographic, vital signs, laborator, medication	DT RF SVM KNN LR	Future studies will assess how early identification of cardiogenic shock and potential effects on prompt treatment may alter patient outcomes
	2021	Bai, Z. [41]	Predictive model of evolution towards CS in patients with STEMI	Demographic, pre-existing diagnoses, ECG, laboratory	LASSO LR SVM DT	Larger groups of patients; blood glucose analysis used as a predictive factor of CS
	2022	Chang, Y. [42]	CS prediction 2 h before the need for the first intervention	Demographic, vital signs, laboratory	XGB MLP TCN	Future studies to include the integration of HF or AMI specific elements to increase accuracy
	2023	Jajcay, N. [43]	Predicting CS in acute coronary syndrome (ACS) patients	Demographic, vital signs, laboratory, ECG	KNN	Superior computational power would include more models for analysis and allow the imputation analysis of analyzing more datasets
	2022	Jentzer, J. C. [44]	Phenotype CS	Laboratory	HC LCA KMCk	Integration of multi-biomarker/imaging (ECG, echocardiography, angiography) to understand the differences in underlying pathophysiology that separate these clinical subphenotypes could improve phenotyping
	2023	Wang, L. [45]	Clinical phenotypes of CS	Demographic and medical history, vital signs, laboratory, treatment	CA	Association between endpoints within individual SCAI stages and ML-derived phenotypes whose aim is to characterize disease severity as it evolves over the course of a hospital stay
	2022	Bohm, A. [46]	Clinical predictive model of progression to CS in patients with ACS	Demographic, vital signs, laboratory	LR	Validation on an external cohort
	2023	Popat, A. [47]	Early prediction of CS in acute heart failure or MI	Demographic, Laboratory	ML	Conducting studies also outside the USA
	2022	Rong, F. [48]	Predicting the 30-day mortality of elderly patients with CS	Demographic, vital sign, laboratory, comorbidities, Echocardiograpy	Cox model LASSO SAPSII	Larger groups of patients
	2023	Mo, Z. [49]	Assessing the prognosis of ECMO treatment in elderly patients with CS	Demographic, vital sign, laboratory, comorbidities, medications	RF DT	A larger batch of patients followed for more than 6 months
Cardiomyopathy	2024	Cau, R. [50]	Differential diagnosis of cardiomyopathy phenotypes	Demographic, vital sign, laboratory, ECG	CNN	Randomized trials are crucial
	2023	Haimovich, J. S. [51]	Differential diagnosis between LV hypertrophy: cardiac amyloidosis and hypertrophic cardiomyopathy	Demographic, vital signs, laboratory, medication, ECG	CNN	Studies on cardiomyopathies to include the athletic heart
	2023	Beneyto, M. [52]	Predicting hypertensive origin in left ventricular hypertrophy (LVH)	Clinical, Laboratory, ECG, echocardiograpy	DT RF SVM	Additional studies from non-tertiary centers
	2022	Eckstein, J. [53]	Diagnosis of cardiac amyloidosis (CA)	Demographic, clinical, echocardiogray, CMR	KNN SVM DT	Multicenter evaluation of patients with early stage cardiac amyloidosis
	2021	Siontis, K. C. [54]	Diagnosis of hypertrophic cardiomyopathy (HCM) in children and adolescents	Demographi, ECG,	CNN	Multicenter studies in children under 5 years
	2020	Ko, W.-Y. [55]	Diagnosis of HCM particularly in younger patients	Demographic, ECG	CNN	Further refinement and external validation
	2022	Hwang, I.-C. [56]	Differential diagnosis of LVH	Echocardiography	CNN	Future studies that include rare LVH etiologies: Fabry disease, Danon syndrome, transthyretin amyloidosis
	2023	Zhou, M. [57]	Differentiating ischemic cardiomyopathy from dilated cardiomyopathy	Echocardiography	RF LR CNN XGB	Multicenter external validation on larger patient batches
	2023	Cau, R. [58]	Diagnosis of Takotsubo cardiomyopathy	Demographic, Echocardiography	RT	Longitudinal and prospective studies to assess predictive performance in different cohorts and validate these findings
	2023	De Filippo, O. [59]	The prediction of prognosis in hospital patients with Takotsubo syndrome	Demographic, ECG, Echocardiograpy, laboratory, medications	LR CA	Multicenter studies beyond European and Asian ethnicities
	2021	Jefferies, J. L. [60]	Predictive screening model for potential patients with Fabry disease	Demographic, clinical, echocardiography, medications, laboratory	ML	Future clinical implementation studies
	2022	Sotto, J. [61]	The prediction of etiology of LVH	ECG Echocardiograpy	CNN	Multicenter studies to identify other causes of ventricular hypertrophy, such as Fabry disease or cardiac amyloidosis
	2023	Zhang, Y. [62]	Diagnosis of arrhythmogenic cardiomyopathy (ACM) and dilated cardiomyopathy (DCM)	Echocardiograpy, Genes	CA RF	Checking for other genes involved in pathogenesis of ACM and DCM
	2022	Papageorgiou, V. E. [63]	Detection of an arrhythmogenic heart disease (ARVD)	ECG	CNN	Checking integration into clinical practice
	2023	Harmon, D.M. [64]	Validation study of cardiac amyloidosis diagnosis	Demographic, ECG	KNN SVM DT CNN	Future multicentric studies to validate the diagnosis in different ethnicities in the presence of left bundle branch block or LVH
	2023	Cotella, J. I. [65]	Measuring ejection fraction and longitudinal strain in cardiac amyloidosis	Echocardiograpy	LR	Studies on larger batches of patients
	2023	Zhang, X. [66]	Non-invasive diagnostic method for myocardial amyloidosis	Echocardiograpy	RF SVM LR	Studies on larger, multicentre patient groups
	2023	Goswami, R. [67]	Predicting death or transplantation of transthyretin amyloid cardiomyopathy (ATTR-CM)	Hemodynamic, clinical, Echocardiograpy	CNN	Larger groups of patients in multicenter studies
	2023	Michalski, A. A. [68]	Diagnosis of Fabry disease	Demographics, clinical, Echocardiograpy, laboratory	NLP	Prospective studies on larger groups of patients with improved NLP
	2023	Jefferies, J. [69]	Predicting the risk of arrhythmias in Fabry disease	Demographics, Clinical data, ECG, Echocardiograpy	ML	Multicentric studies
	2023	Stolpe, G. [70]	The prediction of sudden cardiac death in HCM	Demographic, Clinical data, Echocardiograpy	RF	Definition of sensitivity and specificity
Cardiovascular Imaging	2022	Zhang, X. [71]	A predictive model for classifying HCM, DCM, and healthy patients	CMR	RF	Multicentric studies
	2023	Tatsugami, F. [72]	Prediction of myocardial infarction or cardiac death	CT cardiac	CNN	External validation for cardiac CT on larger datasets
	2022	O’Brien, H. [73]	Diagnosis of myocardial scars	MRI-LGE CT-DE	SVM	Larger studies with larger CT-DE data to establish optimal imaging parameters and characteristics
	2022	Wen, D. [74]	Identification of hemodynamically significant coronary artery stenosis	CCTA FFR	DT	Validation studies on larger groups of patients
	2023	Lara-Hernández, A. [75]	Quantitative myocardial perfusion	CCTA	DL	Extending the method to other dynamic imaging modalities
	2023	Griffin, W.F. [76]	Detection and grading of coronary stenoses	CCTA QCA FFR	ML	Application of the method after the results of the INVICTUS trial
	2022	Bandt, V. [77]	Identify significant CAD pre-TAVR	CCTA	ML	Additional studies on larger batches
	2022	Li, X.-N. [78]	Highlighting the vulnerability of the coronary artery plate	CCTA	ML	Validation studies
	2021	Lyu, T. [79]	Deep learning approach in reducing the radiation dose of CT	CT cardiac	CNN	Additional studies on larger batches
	2023	Zhang, R. [80]	Diagnosis of myocardial perfusion imaging	SPECT-CT	CNN	The larger cohort in the next stage of our study
	2023	Khunte, A. [81]	Detection of left ventricular systolic dysfunction	ECG, Echocardiography	CNN	Use of this algorithm as a screening method for LV systolic dysfunction among individuals with no clinical disease
	2024	Pieszko, K. [82]	Prediction of left atrial appendage thrombus (LAT)	TTE TOE	LR, DT	Studies in different populations to assess the performance and to evaluate performance in specific subgroups based on sex or race
	2023	Liu, Z. [83]	Diagnosis of right ventricular abnormalities	CMR	DNN	Another validation study by comparing with human experts
	2023	Wang, Y. [84]	Improved myocardial strain analysis	CMR	CNN	Larger batches
	2024	Yu, J. [85]	Assessment of LV function	TOE	ML	Larger batches
	2023	Laumer, F. [86]	Differential diagnosis of Takotsubo syndrome (TTS) and acute myocardial infarction (AMI)	TTE	CNN	Future studies on a larger population are to differentiate the two conditions in the acute phase
	2024	Lee, D. [87]	Detecting obstructive CAD	CCTA	DL	Larger batches
	2023	Kapalos, A. [88]	Segmentation of cardiac T1 and T2 mapping images	CMR	DNN	Future work will focus on ensuring the accurate measurement of tissue properties
Congenital Heart Disease	2023	Ishikita, A. [89]	Prediction of adverse cardiovascular events in adults with repaired tetralogy of Fallot	CMR Clinical data	RF	Follow-up studies including multiple centers with longer follow-up
	2023	De Vries, I.R. [90]	Screening of congenital heart disease (CHD)	ECG	ANN	Future work should aim to improve the signal processing chain to omit or reduce the need for combining multiple heartbeats, potentially further allowing for the additional analysis of arrhythmias and better performance
	2021	Lv, J. [91]	Screening and detecting CHD in children	Heart sounds	CNN	Larger patient cohorts
	2022	Majeed, A. [92]	A greater risk of developing executive function deficits in children with complex CHD	Demographic, medical and surgical history, family social class	RF DT	Larger patient cohorts
	2022	Sakai, A. [93]	Detection of small ventricular septal defects	Fetal cardiac ultrasound	DNN	Multicenter joint research.
	2022	Gearhart, A. [94]	Analysis of pediatric echocardiograms	ETT	CNN	Future work could incorporate transfer learning to reduce the manual workload
Electrocardiography	2023	Valente Silva, B. [95]	Diagnosis of pulmonary embolism	ECG	DNN	External samples from other centers
	2023	Adedinsewo, D. [96]	Detecting moderate-to-severe acute cellular rejection (ACR) among heart transplant (HT) recipients	Demographic, clinical characteristic, ECG, TTE	CNN	Future directions would include evaluating the potential benefit of combining AI-ECG screening with blood testing methods, evaluation of the AI-ECG for detecting ACR and antibody-mediated rejection (AMR) combined
	2023	Shiraishi, Y. [97]	Risk of sudden cardiac death	ECG	CNN RNN	Studies on larger batches of patients; future studies will include batches of patients treated with angiotensin receptor-neprilysin inhibitors and sodium-glucose cotransporter 2 inhibitors
	2023	Hirota, N. [98]	The biological age of the patient is associated with cardiovascular events	ECG	CNN	The clinical significance of AgeDiff in patients older than 60 years should be re-evaluated in different cohorts, such as multicenter cohorts or the general population
	2023	Wouters, P. C. [99]	Effects of cardiac resynchronization therapy	ECG	DNN	Prospective studies; the results need to be validated in a patient group that received a CRT-P device
	2023	Raghunath, A. [37]	Onset of atrial fibrillation	ECG	CNN RNN	Future studies with characteristic information for the population (e.g., racial background)
	2023	Liu, C.-W. [100]	Prediction of LVH	ECG	CNN DNN	Analyzing other surrogate end points in cardiovascular diseases (such as left ventricular diastolic dysfunction, new-onset myocardial infarction, or heart failure)
	2023	Zaver, H. [101]	Predicting cardiac events and incident AF in patients who have received a liver transplant	ECG	CNN DNN	Larger patient cohorts
	2023	Naser, J. [102]	Concentration of sex hormones	ECG; Laboratory	CNN	Larger patient cohorts
	2023	Vaid, A. [103]	Valvular diseases	ECG	CNN	Further external validation from another health system
	2023	Jiang, J. [38]	Predicting the risk of recurrence in patients with paroxysmal atrial fibrillation after catheter ablation	ECG	CNN	Further calibration and validation using high-quality prospective studies
Heart Failure	2023	Khan, M. S. [104]	Early diagnosis of HF, stratifying HF disease severity	Demographic, clinical parameters, ECG, TTE	SVM ANN CNN	More datasets are needed for validation and increased transparency through an understanding of AI models
	2023	Almujally, N. A. [105]	Remote monitoring of patients with acute heart failure	Demographic, clinical, laboratory	CNN RNN	The IoT-based system can be further expanded with different types of wearable medical healthcare devices that can be operated on tablets or smartphones
	2022	Kobayashi, M. [106]	Predicting heart failure incidence in asymptomatic individuals	Demographic, TTE, laboratory, medication, cardiovascular risk factors	CA DT	A further prospective multicenter study is needed to assess the applicability
	2020	Segar, M.W. [107]	Phenomapping of patients with HF with preserved ejection fraction (HFpEF)	TTE, Laboratory,	CA	The use of more comprehensive data, as well as a larger number of patient variables, may have yielded different results
	2024	Bourazana, A. [108]	HF diagnosis, monitoring, and management	Clinical examinations, TTE, laboratory	SVM ANN CNN	Issues that remain unresolved concern diagnosis, classification, and treatment. Future studies will assess heart failure with preserved ejection fraction (HFpEF)
	2022	Bachtiger, P. [109]	Screening for HF with reduced ejection fraction	ECG ETT	CNN	Screening for further priority cardiovascular diseases, such as valvular heart pathology, using AI-enabled phonocardiography
	2021	Harmon, D. M. [110]	Diagnosis of HF with reduced ejection fraction (HFrEF)	ECG	CNN	Prospective evaluation of the AI-ECG for ventricular dysfunction for treatment of HF in the acute setting
	2021	Kwon, J.-M. [111]	Early detection of HFpEF	ECG	DLM	The algorithm used must be further validated in patients with HFrEF in other countries
	2024	Wu, J. [112]	Predicting mortality in patients with undiagnosed HFpEF	Demographic, ETT	NLP	Other observational studies
	2023	Akerman, A. P. [113]	Detection of HF	ETT ECG	CNN	Future work is required to guide more transparent and patient-level interpretation
	2021	Pană, M.-A. [114]	Predicting HF exacerbation	Patient’s voice	SVM ANN KNN	Larger batches of patients
	2024	Cheungpasitporn, W. [115]	Treatment for HF patients with acute kidney disease	Demographic, vital signs, laboratory, medication	ML	External validation
	2023	Kamio, T. [116]	Predicting clinical outcomes in acute heart failure	Demographic, vital signs, laboratory, comorbidities, medication	CNN SVM LSVCe XGB	Further research is required to determine the generalizability of these conclusions to other populations and settings
Heart Transplant	2022	Naruka, V. [117]	Predicting graft failure and mortality	Demographic, biomarker, CMR	ANN CNN RF SVM DT	Prospective multicenter studies collecting data on the immunosuppression regime or the causative factors for length of hospital stay were not studied
	2021	Briasoulis, A. [118]	Predicting survival and acute rejection after HT	Clinical and Laboratory	LR DT SVM KNN	Future studies with analysis of other predictors after heart transplantation
	2023	Seraphin, T. P. [119]	Predicting the degree of cellular rejection from pathology	Pathological archive	CNN	Validation in larger cohorts for clinical-grade AI biomarkers
	2022	Ozcan, I. [120]	Predicting major adverse cardiovascular events (MACE) risk post-HT	Clinical and laboratory, ECG	CNN	Further research is to guide screening and treatment strategies for HT patients using this algorithm
	2023	Sharma, S. [121]	Predicting the susceptibility of HT patients to COVID-19	Demographic, Clinical, Laboratory	CNN MLP	Further research and implementation of these technologies in diagnosis, monitoring, and detection
	2020	Glass, C. [122]	Identify myocyte damage in HT acute cellular rejection (ACR)	Comorbidities	CNN	Validation in prospective studies with larger cohorts
	2023	Al-Ani, M. A. [123]	Predicting survival rates in intra-aortic balloon pump (IABP) used as a bridge to HT compared to the Impella	Clinical, Laboratory, Related Disease	XGB	External validation
	2021	Peyster, E. G. [124]	Histological grading of cardiac allograft rejection	Endomyocardial biopsy slides	SVM	External validation
	2022	Lipkova, J. [125]	Assessment of cardiac allograft rejection from	Endomyocardial biopsy slides	CNN	Larger studies
	2023	GIuste, F.O. [126]	Enhancing risk assessment of rare pediatric heart transplant rejection	Heart Biopsy	CNN DNN	Future work will focus on generating additional expert-annotated examples of cellular rejection signs to further improve and validate our model’s performance
	2022	Lisboa, P. J. G. [127]	Survival prediction in heart transplantation	Clinical, Demographic	GANN PRN	Prospective studies
	2022	Ruiz Morales, J. [128]	Predicting outcomes after HT	ECG	CNN	Larger studies
	2020	Agasthi, P. [129]	Predicting 5-year mortality and graft failure in patients with HT	Demographic, clinical	GBM	Larger studies
	2021	Ozcan, I. [130]	Predicting MACE after HT	ECG	CNN	Larger studies
Hypertension	2020	Soh, D. C. K. [131]	Diagnosis of hypertension	ECG	KNN	Future studies with larger cohorts and validation studies
	2020	López-Martínez, F. [132]	Predicting hypertension	Demographic, laboratory, vital signs	ANN	Future studies with new risk factors
	2020	Wu, X. [133]	Clinical prediction of hypertension in young patients	Clinical, comorbidities	XGB	Larger studies
	2020	Aziz, F. [134]	Effectiveness of hypertension treatment	Demographic, clinical, treatment	ANN SVR	Studies on larger batches of patients
	2020	Koshimizu, H [135]	Blood pressure variability	Demographic, vital signs	DNN	Longer-term studies
	2023	Hamoud, B. [136]	Estimating blood pressure	Videos of subjects	CNN	Extending the training set with subjects who are older or estimating other vital signs such as heart rate, oxygen saturation, and body temperature
	2023	Cheng, H. [137]	Blood pressure prediction	IPPG	CNN	Larger studies
	2023	Xing, W. [138]	Hypertension screening and disease prevention	Facial videos	DNN	Larger studies
	2023	Visco, V. [139]	Hypertension prediction and management	Clinical, Laboratory, TTE	ANN SVM KNN	Implementation studies
	2022	Maqsood, S. [140]	Blood pressure estimation and measurement	PPG ECG	DNN	Future studies will combat the “Black Box” because they lack the declarative knowledge necessary to explain the outcomes further
	2023	Herzog, L. [141]	Hypertension management	Demograpic, clinical, Comorbidities	DNN	Validation of results with a randomized controlled clinical trial
	2021	Khthir, R. [142]	Predictive factors for hypertension in patients with type 2 diabetes	Demographic, Clinical data, Laboratory	RF	Larger studies
	2023	Aryal, S. [143]	Evidence that blood pressure is closely correlated with the microbiota	Demographic, clinical, laboratory, genetic tests	XGB RF NB	Larger studies and prospective studies
	2023	Lin, Z. [144]	Prediction of hypokalemia in patients with hypertension	Laboratory, comorbidities, antihypertensive medications	RF	Prospective studies
Pulmonary Hypertension	2020	Kusunose, K. [145]	Confirmation of the need for RHC in patients with suspected pulmonary hypertension (PH)	Clinical Findings CXR ETT PAP	CNN	Further validation is necessary to determine the feasibility of CXR and larger numbers for the differentiation of pre- and post-capillary PH
	2021	Hardacre, C. J. [146]	MRI may allow for reduction of right heart catheterization (RHC)	CMR, laboratory	CNN DT RF	Future studies with AI to diagnose PAH that can be applied to CT
	2022	Ragnarsdottir, H. [147]	Prediction of PH in newborns	ECG	CNN	Larger batches of patients
	2021	Chakravarty, K. [148]	PH therapies for COVID-19 treatment	Clinical, laboratory	RF	Further validation is necessary
	2021	Rahaghi, F. N. [149]	Detection of pulmonary arterial disease	Demographic, PAP, CT imaging, ECG, ETT, Laboratory	CNN	Future studies on the multicenter registry; further work is to determine the specificity and sensitivity of such metrics as well as their value for prognostication and monitoring response to therapeutic intervention
	2022	Shi, B. [150]	Prediction of PH	Biomarkers, Hemodynamics, PAP	DT RF RT LR SVM	Prospective studies on human subjects
	2021	Amodeo, I. [151]	Predict PH in newborns with congenital diaphragmatic hernia	CMR images	CNN SVM	Future prospective multicenter cohort study for validation
	2022	Van der Bijl, P. [152]	Diagnosis of PH	ETT CXR	ANN	studies including patients with tricuspid regurgitation
	2021	Swift, A. J. [153]	Diagnosis of PH	CMR PAP, ETT	SVM LR	Larger cohorts
	2022	Charters, P. F. P. [154]	Predicting survival in patients with suspected PH	ETT, CTPA	ML	Larger studies with larger cohorts
	2022	Fortmeier, V. [155]	Predicting PH in patients with severe TR	ETT PAP demographic, laboratory, comorbidities	XGB	Randomized controlled trials to investigate whether specifically patients with elevations of predicted mPAP still benefit from transcatheter interventions
	2022	Liu, C.-M. [156]	Predicting future risk for cardiovascular mortality in patients with PH	ECG ETT	CNN	Further studies on larger cohorts are necessary for validation
	2023	Lu, W. [157]	Diagnostic biomarkers for idiopathic pulmonary hypertension (IPAH)	Gene	RF	Larger studies
	2023	Yu, X. [158]	PH diagnosis	ETT, PAP CT images	KNN	Larger studies
	2023	Hyde, B. [159]	Distinguishing between patients with PAH and those without PAH at 6 months before diagnosis	Demographics, diagnoses, laboratory, medications	RF	External validation
	2023	Zhang, N. [160]	Diagnosis of PH	ETT, CTPA	SVM XGB	Future studies will explore the volumetric information of heart and pulmonary artery morphology as well as the spatial relationship between different intra- and extra-cardiac structures to improve the accuracy of PAP parameter evaluation
	2024	Hirata, Y. [161]	Predicting PH	RHC	LR	Validation and further research to assess clinical utility in PH diagnosis and treatment decision making
	2024	Imai, S. [162]	PAH detection	CXR PAP	CNN	Future studies should include diseases with CXR images similar to PAH, such as cardiac hypertrophy, and collect data from diverse clinical settings
	2024	Ragnarsdottir, H. [163]	Assessing PH in newborns	ETT	CNN	Future studies could be applied to ECHOs from the adult population, with retraining required
	2024	Dwivedi, K. [164]	Improving PH prognosis by detecting pulmonary fibrosis	Demographic, CT pulmonary angiograms, ETT	CNN	Validation study
	2023	Griffiths, M. [165]	Risk prediction model for pediatric PH	Demographics, imaging, hemodynamics, laboratory, comorbidities	RF	Future studies could be applied to the adult population
	2023	Mamalakis, M. [166]	Diagnosis of PH	CT images	CNN	Larger studies
	2024	Tchuente Foguem, G. [167]	Prognosis of survival of PH	Demographics, ETT	SVM	Larger studies
	2024	Han, P.-L. [168]	Diagnosis of congenital heart disease and associated PAH	CXR, CMR ETT	RF	Larger studies
	2024	Anand, V. [169]	Diagnosis PH	ETT PAP ECG	ML	Larger studies
Infective Endocarditis	2024	Lai, C. K.-C. [170]	Predicting infective endocarditis (IE)	Demographic, clinical, ETT, comorbidities	RF	Multicentric study and prospective studies
	2024	Yi, C. [171]	Common biomarkers in IE and sepsis	Laboratory tests, genes	RF LASSO	Future research will require more extensive datasets and experimental validation. Future studies also demonstrate differences between subtypes of sepsis, including significant variations in the abundance and expression levels of immune cell populations
	2023	Galizzi Fae, I. [172]	Risk stratification of patients hospitalized with IE	Laboratory, imaging, treatment, comorbidities	LR DT	Larger studies
Ischemic Heart Disease	2022	Chen, Z. [173]	Myocardial infarction segmentation	CMR	CNN	Future studies that can differentiate old myocardial scar from acute myocardial infarction
	2021	Rauseo, E. [174]	Diagnosis of chronic ischemic disease	CMR	SVM RF	Further studies to determine whether there is a correlation between the radiomic features and the extent of myocardial scarring
	2021	Liu, W.-C. [175]	Diagnosis of acute myocardial infarction (AMI) at the emergency department (ED)	Clinical, ECG, Laboratory	DL	Future studies on large-scale, multi-institute, prospective, or randomized control studies are necessary to further confirm real-world performance
	2020	Zhao, Y. [176]	Early detection of ST-segment elevated myocardial infarction (STEMI)	ECG	CNN	Future studies should be validated in various ethnics
	2020	Cho, Y. [177]	Diagnosis of AMI	Demographic, ECG, laboratory	CNN	A prospective study specifically designed to confirm the accuracy of data from various wearable or portable ECG devices is warranted to apply the DLA to these devices
	2021	Liu, W.-C. [178]	Detecting AMI	Demographic, ECG, laboratory, angiography	DL LR	Further prospective validation with prehospital and in-hospital ECG tests is needed to confirm the performance of our DLM
	2023	Ciccarelli, M. [11]	Prevention of cardiovascular disease (CAD)	Genetic and epigenetic variables, clinical risk factor	RF	Future studies for the prevention of other pathologies
	2021	Velusamy, D. [179]	Diagnosis and prediction of CAD	Demographic, clinical, laboratory, risk factors	RF SVM KNN	Larger studies
	2021	Muhammad, L. J. [180]	Prediction of CAD	Demographic, clinical, diagnosis	Naive Bayes; SVM RF DT	Future studies on other ethnic groups
	2021	Li, D. [181]	Prediction of CAD	Demographic, clinical, laboratory	ML	Future studies for prediction of other pathologies
	2024	Brendel, J. M. [182]	Assessment of CAD in patients undergoing workup for transcatheter aortic valve replacement (TAVR)	CT angiography, laboratory, ETT, comorbidities	CNN	Multicentric study
	2024	Ihdayhid, A. R. [183]	CAD diagnosis and identification of high-risk plaque	Demographic, CCTA ECG	CNN	Future research is needed to investigate the prognostic impact and value of incorporating deep learning techniques into clinical practice compared to the standard of care
	2024	Uzokov, J. [184]	Diagnosis of ischemic heart disease (IHD)	ECG ETT CCTA	DL	Further management using cloud-based innovative digital technologies and artificial intelligence (AI)
	2024	Abdelrahman, K. [185]	Coronary artery calcium scoring detection and quantification	ECG CT imaging	KNN SVM CNN ANN	Future studies to distinguish between non-coronary calcifications such as valvular calcification and other high-density objects (e.g., metal implants) from coronary calcifications
	2024	Park, M. J. [186]	Predicting coronary occlusion in resuscitated out-of-hospital cardiac arrest (OHCA) patients	ECG, Clinical, Biomarkers	DL	Further validation in larger, prospective studies to establish efficacy across diverse clinical settings
	2024	Alkhamis, M. A. [187]	Predicting in-hospital and 30 days adverse events in ACS	Demographic, clinical, ECG, ETT, CCTA	RF XGB SVM LR	Future studies for prediction of 1-year adverse events in ACS
	2024	Zhu, X.; Xie, B. [188]	Prediction of death in patients with first AMI	Demographic Clinical Laboratory ECG	LR, RF XGB SVM MLP	Validating studies on other ethnic groups
	2024	Kasim, S. [189]	Predicting outcomes in non-ST-segment elevation myocardial infarction (NSTEMI) or unstable angina (UA)	Demographic medication	XGB SVM NB RF GLM	Continuous development, testing, and validation of these ML algorithms hold the promise of enhanced risk stratification, thereby revolutionizing future management strategies and patient outcomes
	2023	Oliveira, M. [190]	AMI mortality prediction	Demographic laboratory	LR DT RF XGM SVM	Further studies should be conducted and consider the inclusion of more variables that may be relevant in predicting AMI mortality, such as socioeconomic factors, systolic blood pressure, heart rate, and electrocardiogram results
	2021	Bai, Z. [41]	Prediction for CS risk in patients with STEMI	Demographic ECG laboratory in-hospital events	LR SVM XGB LASSO	Future studies with other risk factor analyses; for example, glycemia is not routinely recorded in our patients and therefore could not be tested as a potential predictor of CS. Further investigations using larger populations are warranted to fully evaluate the applicability of this model
	2024	Azdaki, N. [191]	Predicting CAD in hospital patients	Demographic Clinical comorbidities	ANN	Larger studies on ethnic groups
Pericardial Disease	2024	Zhan, W. [192]	Prognostic model based on malignant pericardial effusion (PE)	genes	LASSO	Future studies to discover immunotherapy drugs
	2022	Liu, Y.-L. [193]	Diagnosis of acute pericarditis and differentiate it from STEMI in the ED	ECG	XGB	Prospective, multicenter study; large-scale, further external validation
	2023	Cheng, C.-Y. [194]	Detecting and measurement of PE	TTE	CNN	Future studies should utilize a multicenter design with greater heterogeneity in the dataset. Further research should evaluate the collapsibility of the cardiac chambers and the presence of tamponade signs
	2022	Wilder-Smith, A. J. [195]	Detection and segmentation of PE	CT images	CNN	Future studies to include CT images of older machines, as this study was performed on state-of-the-art CT
	2022	Piccini, J. P. [196]	Predicting PE after leadless pacemaker implantation	Demographic clinical comorbidities	LASSO LR	A larger number of patients
Peripheral Arterial Disease	2024	McBane, R. D. [197]	Diagnosis patients with peripheral artery disease (PAD) at greatest risk for major adverse events	Demographic Comorbidities Ankle-brachial index	CNN	Future studies to assess the tolerance of such signal acquisition would be important. Further validation studies are required to assess test accuracy and reproducibility in community settings outside of a large-volume academic vascular laboratory
	2024	Rusinovich, Y. [198]	Identifying and classifying the anatomical patterns of PAD	Angiograms	ML	A larger number of patients
	2024	Sasikala, P. [199]	Prediction of PAD and accurate categorization of its severity levels	Demographic clinical angiograms	DT	Future studies for validation
	2024	Li, B. [200]	Prognosis of PAD	Laboratory, Demographic, Comorbidities Treatment	ML	Future validation at other institutions is needed to demonstrate the generalizability of this model
	2024	Masoumi Shahrbabak, S. [201]	Diagnosis of PAD	Arterial pulse waveforms ankle-brachial index BP	DL	External validation.
	2024	McBane, R. D., II [202]	Prediction of major adverse outcomes among patients with diabetes mellitus and PAD	Posterior tibial arterial Doppler waveforms	DNN	Larger studies
	2024	Li, B. [203]	Predict outcomes after infra-inguinal bypass in patients with PAD	Demographic Clinical complications	XGB LR	Larger studies
	2024	Liu, L. [204]	Prediction of PAD in diabetes mellitus (T2DM)	demographic diagnoses, biochemical index test	DT LR RF SVM XGB	Future studies in other regions also need to address the lack of collected and analyzed data on patients’ subjective descriptions, such as their duration of diabetes and smoking habits, which have been reported as associated with PVD in T2DM
Thromboembolic Disease	2024	Nassour, N. [205]	Predicting venous thromboembolism (VTE) in ankle fracture patients	Demographic CXR CT images ultrasonograpy	RF	Studies on larger groups of patients
	2024	Chen, R. [206]	Predicting diagnosis and 1-year risk of VTE	Demographics, laboratory, medications	ML	Future research should focus on refining and validating these models in different healthcare settings as well as informing personalized treatment strategies and exploring their potential utility in predicting VTE recurrence
	2024	Pan, S. [207]	VTE risk prediction for surgical patients	Demographic clinical laboratory comorbidities	ANN LR	Validation studies
	2024	Grdinic, A. G. [208]	Bleeding prediction in patients with cancer-associated thrombosis	Clinical biochemistry diagnosis	LR RF XGB	Validation studies
	2023	Chiasakul, T. [209]	Prediction of VTE	Ultrasonography	LR	Future studies should focus on transparent reporting, external validation, and clinical application of these models
	2023	Wang, X. [210]	Predicting the risk of DVT after knee/hip arthroplasty	Demographic clinical comorbidities	NLP XGB RF SVM RL	In future studies, model accuracy will be further evaluated by performing prospective and real-time predictions of DVT
	2023	Wang, K. Y. [211]	Identification of patients at risk of VTE following posterior lumbar fusion	Demographic, clinical comorbidities	LR XGB	Future studies should seek to externally validate these predictive tools and should examine the potential cost savings provided by predictive analytics models which can accurately identify patients at risk of VTE following spine surgery
	2023	Muñoz, A. J. [212]	Predicting 6-month VTE recurrence in patients with cancer	Demographic, clinical comorbidities	LR DT RF NLP	Future studies are needed to assess the validity of these results
	2021	Razzaq, M. [213]	Predicting the risk of recurrent VTE by biomarkers	Laboratory	ANN	Further experimental validations for these biomarkers
	2023	Valente Silva, B. [95]	Acute pulmonary embolism (PE) diagnosis	ECG laboratory	DNN	Further application in larger cohorts and external validation of the deep learning model are essential to fully validate its performance. These results should be validated in an external sample from other centers in the future, as well as in high-risk patient subgroups, for example, patients with hemodynamic instability
	2022	Contreras-Luján, E. E [214]	Early diagnosis of DVT	Clinical ultrasonography	KNN DT SVM RF	External validation
	2023	Seo, J. W. [215]	Detecting iliofemoral DVT	CT angiography	CNN	Future studies to extend the detection ranges to the infrapopliteal vein for investigating DVT
Valvular Heart Disease	2023	Alhwiti, T. [216]	Predicting in-hospital mortality post-TAVR	Demographic disease	LR	Future studies looking at long-term mortality post-TAVR
	2023	Strange, G. [217]	Identifying severe aortic stenosis (AS) phenotypes associated with high mortality	TTE	ML	Future studies in other geographic regions/health systems or specific ethnic groups
	2023	Ueda, D. [218]	Screening for heart valve disease	CXR TTE	CNN	Further validation with prospectively acquired test datasets from cohorts with various disease prevalences
	2024	Singh, S. [219]	Valvular heart disease screening	TTE	SVM LR CNN	Future studies to validate these data
	2024	Brown, K. [220]	Screening for rheumatic heart disease (RHD) in children	TTE	CNN	Future work needs to be pursued incorporating other features of RHD into our current AI algorithms as well as feasibility testing for field implementation in RHD endemic regions
	2024	Toggweile, S. [221]	TAVR planning and implantation	Cardiac CT	CNN	Further studies are required to demonstrate if these results can be reproduced by other centers using different scanners and different protocols for image acquisition (external validation)
	2021	Solomun M.D. [222]	Screening of AS	ECG	NLP	Future studies leveraging NLP-derived data to evaluate the association between the severity of AS and clinical outcomes, along with identifying predictors of AS progression
	2022	Aoyama, G. [223]	Diagnosis of AS	Cardiac CT	DNN	In future work, we should compare the intraoperative direct observation and the manual measurements by physicians with the automatic measurements by our proposed method to more concretely verify the clinical effectiveness and risks of the proposed method
	2024	Dasi, A. [224]	Predicting post-transcatheter aortic valve replacement	TTE Cardiac CT	ANN SVR	Future studies can focus on building upon these AI models to account for the nonlinear and complex relationships among postoperative AV pressure gradient, AVA, and patient outcomes
	2023	Krishna, H. [225]	Assessment of AS	ETT	ANN	Future work will aim to utilize AI to categorize AS severity, incorporating multiple echocardiographic variables
	2024	Xie, L.-F. [226]	Predicting postoperative adverse outcomes following surgical treatment of acute type A aortic dissection	Demographic, clinical	LASSO XGB	Multicenter study; external validation data are needed to test the clinical utility of the model
	2024	Zhou, M. [227]	Screening of aortic dissection	ECG	CNN	Larger studies; future prospective studies could enhance the efficacy of this AI model
	2023	Irtyuga, O [228]	Screening of AS in patients with and without bicuspid aortic valve	TTE	SVM ANN RF DR	Future research efforts should focus on early diagnostics and strategies to delay the progression of degenerative aortic valve disease
	2023	Kennedy, L. [229]	Prediction of the mechanical function of thoracic aortic aneurysm (TAA) tissue	TTE medical history genetic paneling	SVM GPR	Larger studies and an investigation are needed to compare the accuracy of risk levels assigned by the integrated ML approach compared to that of diameter thresholding using histopathological classification of tissue

## Data Availability

Not applicable.

## Abbreviations

ACM	arrhythmogenic cardiomyopathy
ACR	acute cellular rejection
ACS	acute coronary syndrome
AF	atrial fibrillation
AI	artificial intelligence
AI-QCT	artificial intelligence-enabled quantitative coronary computed tomography
AMI	acute myocardial infarction
ARVD	arrhythmogenic heart disease
ATTR-CM	transthyretin amyloid cardiomyopathy
CCTA	coronary computed tomography angiography
CMR	cardiovascular magnetic resonance
CNN	convolutional neural network
DL	deep learning
DCM	dilated cardiomyopathy
DECT	dual-energy computed tomography
ECG	electrocardiogram
FFR	fractional flow reserve
HCA	hierarchical
KMCk	means clustering
IABP	intra-aortic balloon pump
ICA	invasive coronary angiography
IE	infective endocarditis
LA	left atrium
LAAT	left atrial appendage thrombus
LCA	latent class analysis
LV	left ventricle
LVH	left ventricular hypertrophy
MACE	major adverse cardiovascular events
MAPSE	mitral annular plane systolic excursion
MI	myocardial infarction
ML	machine learning
MLP	multiple layer perceptron
MRI	magnetic resonance imagining
PAP	pulmonary artery pressure
PCAT	per coronary adipose tissue
PPG	photoplethysmography
QCA	quantitative coronary angiography
RA	right atrium
RV	right ventricle
STEMI	ST-elevation myocardial infarction
TAVR	transcatheter aortic valve replacement
TTE	transthoracic echocardiography
TOE	transesophageal echocardiography
TCN	temporal convolutional network
XCB	machine learning model based on the xgboost

## References

[1] Beam A.L., Drazen J.M., Kohane I.S., Leong T.-Y., Manrai A.K., Rubin E.J. (2023). Artificial Intelligence in Medicine. N. Engl. J. Med..

[2] Lindstrom M., DeCleene N., Dorsey H., Fuster V., Johnson C.O., LeGrand K.E., Mensah G.A., Razo C., Stark B., Turco J.V. (2022). Global Burden of Cardiovascular Diseases and Risks Collaboration, 1990–2021. J Am Coll Cardiol..

[3] Gala D., Behl H., Shah M., Makaryus A.N. (2024). The Role of Artificial Intelligence in Improving Patient Outcomes and Future of Healthcare Delivery in Cardiology: A Narrative Review of the Literature. Healthcare.

[4] Sun X., Yin Y., Yang Q., Huo T. (2023). Artificial Intelligence in Cardiovascular Diseases: Diagnostic and Therapeutic Perspectives. Eur. J. Med. Res..

[5] Johnson K.W., Torres Soto J., Glicksberg B.S., Shameer K., Miotto R., Ali M., Ashley E., Dudley J.T. (2018). Artificial Intelligence in Cardiology. J. Am. Coll. Cardiol..

[6] Shehab M., Abualigah L., Shambour Q., Abu-Hashem M.A., Shambour M.K.Y., Alsalibi A.I., Gandomi A.H. (2022). Machine Learning in Medical Applications: A Review of State-of-the-Art Methods. Comput. Biol. Med..

[7] Yoon C.H., Torrance R., Scheinerman N. (2022). Machine Learning in Medicine: Should the Pursuit of Enhanced Interpretability Be Abandoned?. J. Med. Ethics.

[8] Kahr M., Kovács G., Loinig M., Brückl H. (2022). Condition Monitoring of Ball Bearings Based on Machine Learning with Synthetically Generated Data. Sensors.

[9] Mosqueira-Rey E., Hernández-Pereira E., Alonso-Ríos D., Bobes-Bascarán J., Fernández-Leal Á. (2023). Human-in-the-Loop Machine Learning: A State of the Art. Artif. Intell. Rev..

[10] Cho H., Keenan G., Madandola O.O., Dos Santos F.C., Macieira T.G.R., Bjarnadottir R.I., Priola K.J.B., Dunn Lopez K. (2022). Assessing the Usability of a Clinical Decision Support System: Heuristic Evaluation. JMIR Hum. Factors.

[11] Ciccarelli M., Giallauria F., Carrizzo A., Visco V., Silverio A., Cesaro A., Calabrò P., De Luca N., Mancusi C., Masarone D. (2023). Artificial Intelligence in Cardiovascular Prevention: New Ways Will Open New Doors. J. Cardiovasc. Med..

[12] Busnatu Ș., Niculescu A.-G., Bolocan A., Petrescu G.E.D., Păduraru D.N., Năstasă I., Lupușoru M., Geantă M., Andronic O., Grumezescu A.M. (2022). Clinical Applications of Artificial Intelligence—An Updated Overview. J. Clin. Med..

[13] Van den Eynde J., Lachmann M., Laugwitz K.-L., Manlhiot C., Kutty S. (2023). Successfully Implemented Artificial Intelligence and Machine Learning Applications in Cardiology: State-of-the-Art Review. Trends Cardiovasc. Med..

[14] Visco V., Ferruzzi G.J., Nicastro F., Virtuoso N., Carrizzo A., Galasso G., Vecchione C., Ciccarelli M. (2021). Artificial Intelligence as a Business Partner in Cardiovascular Precision Medicine: An Emerging Approach for Disease Detection and Treatment Optimization. Curr. Med. Chem..

[15] Soori M., Arezoo B., Dastres R. (2023). Machine Learning and Artificial Intelligence in CNC Machine Tools, A Review. Sustain. Manuf. Serv. Econ..

[16] Javaid M., Haleem A., Pratap Singh R., Suman R., Rab S. (2022). Significance of Machine Learning in Healthcare: Features, Pillars and Applications. Int. J. Intell. Netw..

[17] Goodswen S.J., Barratt J.L.N., Kennedy P.J., Kaufer A., Calarco L., Ellis J.T. (2021). Machine Learning and Applications in Microbiology. FEMS Microbiol. Rev..

[18] Ahmad A.A., Polat H. (2023). Prediction of Heart Disease Based on Machine Learning Using Jellyfish Optimization Algorithm. Diagnostics.

[19] Alzubaidi L., Bai J., Al-Sabaawi A., Santamaría J., Albahri A.S., Al-dabba B.S.N., Fadhel M.A., Manoufali M., Zhang J., Al-Timemy A.H. (2023). A Survey on Deep Learning Tools Dealing with Data Scarcity: Definitions, Challenges, Solutions, Tips, and Applications. J. Big Data.

[20] Srivani M., Murugappan A., Mala T. (2023). Cognitive Computing Technological Trends and Future Research Directions in Healthcare—A Systematic Literature Review. Artif. Intell. Med..

[21] Vinny P.W., Vishnu V.Y., Padma Srivastava M.V. (2021). Artificial Intelligence Shaping the Future of Neurology Practice. Med. J. Armed Forces India.

[22] Zhu R., Jiang C., Wang X., Wang S., Zheng H., Tang H. (2020). Privacy-Preserving Construction of Generalized Linear Mixed Model for Biomedical Computation. Bioinformatics.

[23] Yadav R.S. (2020). Data Analysis of COVID-2019 Epidemic Using Machine Learning Methods: A Case Study of India. Int. J. Inf. Technol..

[24] Hügle M., Omoumi P., van Laar J.M., Boedecker J., Hügle T. (2020). Applied Machine Learning and Artificial Intelligence in Rheumatology. Rheumatol. Adv. Pract..

[25] Sharma A., Pal T., Jaiswal V. (2022). Heart Disease Prediction Using Convolutional Neural Network. Cardiovascular and Coronary Artery Imaging.

[26] Teuwen J., Moriakov N. (2020). Handbook of Medical Image Computing and Computer Assisted Intervention.

[27] Xiong Z., Nash M.P., Cheng E., Fedorov V.V., Stiles M.K., Zhao J. (2018). ECG Signal Classification for the Detection of Cardiac Arrhythmias Using a Convolutional Recurrent Neural Network. Physiol. Meas..

[28] Williams S., Layard Horsfall H., Funnell J.P., Hanrahan J.G., Khan D.Z., Muirhead W., Stoyanov D., Marcus H.J. (2021). Artificial Intelligence in Brain Tumour Surgery—An Emerging Paradigm. Cancers.

[29] Mahesh B. (2020). Machine learning algorithms-a review. Int. J. Sci. Res..

[30] Sarker I.H. (2021). Machine Learning: Algorithms, Real-World Applications and Research Directions. SN Comput. Sci..

[31] Al-Sayed A., Khayyat M.M., Zamzami N. (2023). Predicting Heart Disease Using Collaborative Clustering and Ensemble Learning Techniques. Appl. Sci..

[32] Sahlab N., Sonji I., Weyrich M. (2023). Graph-Based Association Rule Learning for Context-Based Health Monitoring to Enable User-Centered Assistance. Artif. Intell. Med..

[33] Kumar K.A., Gowri S., David J.J.W., Bevish Jinila Y. (2022). An Efficient Association Rule Mining from Distributed Medical Database for Predicting Heart Disease. Proceedings of the 2022 6th International Conference on Computing Methodologies and Communication (ICCMC).

[34] Chaudhuri A.K., Das A., Addy M. (2021). Identifying the Association Rule to Determine the Possibilities of Cardio Vascular Diseases (CVD). Advances in Intelligent Systems and Computing.

[35] Tran K.-V., Filippaios A., Noorishirazi K., Ding E. (2023). False Atrial Fibrillation Alerts from Smartwatches Are Associated with Decreased Perceived Physical Well-Being and Confidence in Chronic Symptoms Management. Cardiol. Cardiovasc. Med..

[36] Baj G., Gandin I., Scagnetto A., Bortolussi L., Cappelletto C., Di Lenarda A., Barbati G. (2023). Comparison of Discrimination and Calibration Performance of ECG-Based Machine Learning Models for Prediction of New-Onset Atrial Fibrillation. BMC Med. Res. Methodol..

[37] Raghunath A., Nguyen D.D., Schram M., Albert D., Gollakota S., Shapiro L., Sridhar A.R. (2023). Artificial Intelligence–Enabled Mobile Electrocardiograms for Event Prediction in Paroxysmal Atrial Fibrillation. Cardiovasc. Digit. Health J..

[38] Jiang J., Deng H., Liao H., Fang X., Zhan X., Wei W., Wu S., Xue Y. (2023). An Artificial Intelligence-Enabled ECG Algorithm for Predicting the Risk of Recurrence in Patients with Paroxysmal Atrial Fibrillation after Catheter Ablation. J. Clin. Med..

[39] Bai Y., Wang Z.-Z., Zhang G.-G., Guo S.-D., Rivera-Caravaca J.M., Wang Y.-L., Jin Y.-Y., Liu Y. (2021). Validating Scores Predicting Atrial Fibrillation Recurrence Post Catheter Ablation in Patients with Concurrent Atrial Fibrillation and Pulmonary Diseases. Ann. Palliat. Med..

[40] Rahman F., Finkelstein N., Alyakin A., Gilotra N.A., Trost J., Schulman S.P., Saria S. (2022). Using Machine Learning for Early Prediction of Cardiogenic Shock in Patients with Acute Heart Failure. J. Soc. Cardiovasc. Angiogr. Interv..

[41] Bai Z., Hu S., Wang Y., Deng W., Gu N., Zhao R., Zhang W., Ma Y., Wang Z., Liu Z. (2021). Development of a Machine Learning Model to Predict the Risk of Late Cardiogenic Shock in Patients with ST-Segment Elevation Myocardial Infarction. Ann. Transl. Med..

[42] Chang Y., Antonescu C., Ravindranath S., Dong J., Lu M., Vicario F., Wondrely L., Thompson P., Swearingen D., Acharya D. (2022). Early Prediction of Cardiogenic Shock Using Machine Learning. Front. Cardiovasc. Med..

[43] Jajcay N., Bezak B., Segev A., Matetzky S., Jankova J., Spartalis M., El Tahlawi M., Guerra F., Friebel J., Thevathasan T. (2023). Data Processing Pipeline for Cardiogenic Shock Prediction Using Machine Learning. Front. Cardiovasc. Med..

[44] Jentzer J.C., Rayfield C., Soussi S., Berg D.D., Kennedy J.N., Sinha S.S., Baran D.A., Brant E., Mebazaa A., Billia F. (2022). Machine Learning Approaches for Phenotyping in Cardiogenic Shock and Critical Illness. JACC Adv..

[45] Wang L., Zhang Y., Yao R., Chen K., Xu Q., Huang R., Mao Z., Yu Y. (2023). Identification of Distinct Clinical Phenotypes of Cardiogenic Shock Using Machine Learning Consensus Clustering Approach. BMC Cardiovasc. Disord..

[46] Bohm A., Jajcay N., Jankova J., Petrikova K., Bezak B. (2022). Artificial Intelligence Model for Prediction of Cardiogenic Shock in Patients with Acute Coronary Syndrome. Eur. Heart J. Acute Cardiovasc. Care.

[47] Popat A., Yadav S., Patel S.K., Baddevolu S., Adusumilli S., Rao Dasari N., Sundarasetty M., Anand S., Sankar J., Jagtap Y.G. (2023). Artificial Intelligence in the Early Prediction of Cardiogenic Shock in Acute Heart Failure or Myocardial Infarction Patients: A Systematic Review and Meta-Analysis. Cureus.

[48] Rong F., Xiang H., Qian L., Xue Y., Ji K., Yin R. (2022). Machine Learning for Prediction of Outcomes in Cardiogenic Shock. Front. Cardiovasc. Med..

[49] Mo Z., Lu Z., Tang X., Lin X., Wang S., Zhang Y., Huang Z. (2023). Construction and Evaluation of Prognostic Models of ECMO in Elderly Patients with Cardiogenic Shock Based on BP Neural Network, Random Forest, and Decision Tree. Am. J. Transl. Res..

[50] Cau R., Pisu F., Suri J.S., Montisci R., Gatti M., Mannelli L., Gong X., Saba L. (2024). Artificial Intelligence in the Differential Diagnosis of Cardiomyopathy Phenotypes. Diagnostics.

[51] Haimovich J.S., Diamant N., Khurshid S., Di Achille P., Reeder C., Friedman S., Singh P., Spurlock W., Ellinor P.T., Philippakis A. (2023). Artificial Intelligence-Enabled Classification of Hypertrophic Heart Diseases Using Electrocardiograms. Cardiovasc. Digit. Health J..

[52] Beneyto M., Ghyaza G., Cariou E., Amar J., Lairez O. (2023). Development and Validation of Machine Learning Algorithms to Predict Posthypertensive Origin in Left Ventricular Hypertrophy. Arch. Cardiovasc. Dis..

[53] Eckstein J., Moghadasi N., Körperich H., Weise Valdés E., Sciacca V., Paluszkiewicz L., Burchert W., Piran M. (2022). A Machine Learning Challenge: Detection of Cardiac Amyloidosis Based on Bi-Atrial and Right Ventricular Strain and Cardiac Function. Diagnostics.

[54] Siontis K.C., Liu K., Bos J.M., Attia Z.I., Cohen-Shelly M., Arruda-Olson A.M., Zanjirani Farahani N., Friedman P.A., Noseworthy P.A., Ackerman M.J. (2021). Detection of Hypertrophic Cardiomyopathy by an Artificial Intelligence Electrocardiogram in Children and Adolescents. Int. J. Cardiol..

[55] Ko W.-Y., Siontis K.C., Attia Z.I., Carter R.E., Kapa S., Ommen S.R., Demuth S.J., Ackerman M.J., Gersh B.J., Arruda-Olson A.M. (2020). Detection of Hypertrophic Cardiomyopathy Using a Convolutional Neural Network-Enabled Electrocardiogram. J. Am. Coll. Cardiol..

[56] Hwang I.-C., Choi D., Choi Y.-J., Ju L., Kim M., Hong J.-E., Lee H.-J., Yoon Y.E., Park J.-B., Lee S.-P. (2022). Differential Diagnosis of Common Etiologies of Left Ventricular Hypertrophy Using a Hybrid CNN-LSTM Model. Sci. Rep..

[57] Zhou M., Deng Y., Liu Y., Su X., Zeng X. (2023). Echocardiography-Based Machine Learning Algorithm for Distinguishing Ischemic Cardiomyopathy from Dilated Cardiomyopathy. BMC Cardiovasc. Disord..

[58] Cau R., Pisu F., Porcu M., Cademartiri F., Montisci R., Bassareo P., Muscogiuri G., Amadu A., Sironi S., Esposito A. (2023). Machine Learning Approach in Diagnosing Takotsubo Cardiomyopathy: The Role of the Combined Evaluation of Atrial and Ventricular Strain, and Parametric Mapping. Int. J. Cardiol..

[59] De Filippo O., Cammann V.L., Pancotti C., Di Vece D., Silverio A., Schweiger V., Niederseer D., Szawan K.A., Würdinger M., Koleva I. (2023). Machine Learning-based Prediction of In-hospital Death for Patients with Takotsubo Syndrome: The InterTAK-ML Model. Eur. J. Heart Fail..

[60] Jefferies J.L., Spencer A.K., Lau H.A., Nelson M.W., Giuliano J.D., Zabinski J.W., Boussios C., Curhan G., Gliklich R.E., Warnock D.G. (2021). A New Approach to Identifying Patients with Elevated Risk for Fabry Disease Using a Machine Learning Algorithm. Orphanet J. Rare Dis..

[61] Soto J.T., Weston Hughes J., Sanchez P.A., Perez M., Ouyang D., Ashley E.A. (2022). Multimodal Deep Learning Enhances Diagnostic Precision in Left Ventricular Hypertrophy. Eur. Heart J. Digit. Health.

[62] Zhang Y., Xie J., Wu Y., Zhang B., Zhou C., Gao X., Xie X., Li X., Yu J., Wang X. (2023). Novel Algorithm for Diagnosis of Arrhythmogenic Cardiomyopathy and Dilated Cardiomyopathy: Key Gene Expression Profiling Using Machine Learning. J. Gene Med..

[63] Papageorgiou V.E., Zegkos T., Efthimiadis G., Tsaklidis G. (2022). Analysis of Digitalized ECG Signals Based on Artificial Intelligence and Spectral Analysis Methods Specialized in ARVC. Int. J. Numer. Method. Biomed. Eng..

[64] Harmon D.M., Mangold K., Baez Suarez A., Scott C.G., Murphree D.H., Malik A., Attia Z.I., Lopez-Jimenez F., Friedman P.A., Dispenzieri A. (2023). Postdevelopment Performance and Validation of the Artificial Intelligence-Enhanced Electrocardiogram for Detection of Cardiac Amyloidosis. JACC Adv..

[65] Cotella J.I., Slivnick J.A., Sanderson E., Singulane C., O’Driscoll J., Asch F.M., Addetia K., Woodward G., Lang R.M. (2023). Artificial Intelligence Based Left Ventricular Ejection Fraction and Global Longitudinal Strain in Cardiac Amyloidosis. Echocardiography.

[66] Zhang X., Liang T., Su C., Qin S., Li J., Zeng D., Cai Y., Huang T., Wu J. (2023). Deep Learn-Based Computer-Assisted Transthoracic Echocardiography: Approach to the Diagnosis of Cardiac Amyloidosis. Int. J. Cardiovasc. Imaging.

[67] Goswami R., Jang J., Ruiz J., Desai S., Paghdar S., Malkani S., Yip D., Leoni J., Patel P., Lyle M. (2023). (28) Artificial Intelligence to Predict Death or Transplant in ATTR Amyloidosis Cardiomyopathy. J. Heart Lung Transplant..

[68] Michalski A.A., Lis K., Stankiewicz J., Kloska S.M., Sycz A., Dudziński M., Muras-Szwedziak K., Nowicki M., Bazan-Socha S., Dabrowski M.J. (2023). Supporting the Diagnosis of Fabry Disease Using a Natural Language Processing-Based Approach. J. Clin. Med..

[69] Jefferies J., Aguiar P., Biondetti G., Warnock D., Kallish S., Nelson M., Giuliano J., Zabinksi J., Boussios C., Curhan G. (2023). (751) Estimation of Arrhythmia Risk in Patients with Fabry Disease Using a Machine Learning Model. J. Heart Lung Transplant.

[70] Stolpe G., Didier R., Martel H., Claire L., Michel N., Sellami S., Essayagh B., Réant P., Donal E., Habib G. (2023). Contribution of Artificial Intelligence and Left Atrial Strain in the Prediction of Sudden Cardiac Death in Hypertrophic Cardiomyopathy. Results of a Multicentric Cohort. Arch. Cardiovasc. Dis. Suppl..

[71] Zhang X., Cui C., Zhao S., Xie L., Tian Y. (2022). Cardiac Magnetic Resonance Radiomics for Disease Classification. Eur. Radiol..

[72] Tatsugami F., Nakaura T., Yanagawa M., Fujita S., Kamagata K., Ito R., Kawamura M., Fushimi Y., Ueda D., Matsui Y. (2023). Recent Advances in Artificial Intelligence for Cardiac CT: Enhancing Diagnosis and Prognosis Prediction. Diagn. Interv. Imaging.

[73] O’Brien H., Williams M.C., Rajani R., Niederer S. (2022). Radiomics and Machine Learning for Detecting Scar Tissue on CT Delayed Enhancement Imaging. Front. Cardiovasc. Med..

[74] Wen D., Xu Z., An R., Ren J., Jia Y., Li J., Zheng M. (2022). Predicting Haemodynamic Significance of Coronary Stenosis with Radiomics-Based Pericoronary Adipose Tissue Characteristics. Clin. Radiol..

[75] Lara-Hernández A., Rienmüller T., Juárez I., Pérez M., Reyna F., Baumgartner D., Makarenko V.N., Bockeria O.L., Maksudov M., Rienmüller R. (2023). Deep Learning-Based Image Registration in Dynamic Myocardial Perfusion CT Imaging. IEEE Trans. Med. Imaging.

[76] Griffin W.F., Choi A.D., Riess J.S., Marques H., Chang H.-J., Choi J.H., Doh J.-H., Her A.-Y., Koo B.-K., Nam C.-W. (2023). AI Evaluation of Stenosis on Coronary CTA, Comparison with Quantitative Coronary Angiography and Fractional Flow Reserve. JACC Cardiovasc. Imaging.

[77] Brandt V., Schoepf U.J., Aquino G.J., Bekeredjian R., Varga-Szemes A., Emrich T., Bayer R.R., Schwarz F., Kroencke T.J., Tesche C. (2022). Impact of Machine-Learning-Based Coronary Computed Tomography Angiography–Derived Fractional Flow Reserve on Decision-Making in Patients with Severe Aortic Stenosis Undergoing Transcatheter Aortic Valve Replacement. Eur. Radiol..

[78] Li X.-N., Yin W.-H., Sun Y., Kang H., Luo J., Chen K., Hou Z.-H., Gao Y., Ren X.-S., Yu Y.-T. (2022). Identification of Pathology-Confirmed Vulnerable Atherosclerotic Lesions by Coronary Computed Tomography Angiography Using Radiomics Analysis. Eur. Radiol..

[79] Lyu T., Zhao W., Zhu Y., Wu Z., Zhang Y., Chen Y., Luo L., Li S., Xing L. (2021). Estimating Dual-Energy CT Imaging from Single-Energy CT Data with Material Decomposition Convolutional Neural Network. Med. Image Anal..

[80] Zhang R., Wang P., Bian Y., Fan Y., Li J., Liu X., Shen J., Hu Y., Liao X., Wang H. (2023). Establishment and Validation of an AI-Aid Method in the Diagnosis of Myocardial Perfusion Imaging. BMC Med. Imaging.

[81] Khunte A., Sangha V., Oikonomou E.K., Dhingra L.S., Aminorroaya A., Mortazavi B.J., Coppi A., Brandt C.A., Krumholz H.M., Khera R. (2023). Detection of Left Ventricular Systolic Dysfunction from Single-Lead Electrocardiography Adapted for Portable and Wearable Devices. NPJ Digit. Med..

[82] Pieszko K., Hiczkiewicz J., Łojewska K., Uziębło-Życzkowska B., Krzesiński P., Gawałko M., Budnik M., Starzyk K., Wożakowska-Kapłon B., Daniłowicz-Szymanowicz L. (2024). Artificial Intelligence in Detecting Left Atrial Appendage Thrombus by Transthoracic Echocardiography and Clinical Features: The Left Atrial Thrombus on Transoesophageal Echocardiography (LATTEE) Registry. Eur. Heart J..

[83] Liu Z., Li H., Li W., Zhang F., Ouyang W., Wang S., Zhi A., Pan X. (2023). Development of an Expert-Level Right Ventricular Abnormality Detection Algorithm Based on Deep Learning. Interdiscip. Sci..

[84] Wang Y., Sun C., Ghadimi S., Auger D.C., Croisille P., Viallon M., Mangion K., Berry C., Haggerty C.M., Jing L. (2023). StrainNet: Improved Myocardial Strain Analysis of Cine MRI by Deep Learning from DENSE. Radiol. Cardiothorac. Imaging.

[85] Yu J., Taskén A.A., Flade H.M., Skogvoll E., Berg E.A.R., Grenne B., Rimehaug A., Kirkeby-Garstad I., Kiss G., Aakhus S. (2024). Automatic Assessment of Left Ventricular Function for Hemodynamic Monitoring Using Artificial Intelligence and Transesophageal Echocardiography. J. Clin. Monit. Comput..

[86] Laumer F., Di Vece D., Cammann V.L., Würdinger M., Petkova V., Schönberger M., Schönberger A., Mercier J.C., Niederseer D., Seifert B. (2022). Assessment of Artificial Intelligence in Echocardiography Diagnostics in Differentiating Takotsubo Syndrome from Myocardial Infarction. JAMA Cardiol..

[87] Lee D.-Y., Chang C.-C., Ko C.-F., Lee Y.-H., Tsai Y.-L., Chou R.-H., Chang T.-Y., Guo S.-M., Huang P.-H. (2024). Artificial Intelligence Evaluation of Coronary Computed Tomography Angiography for Coronary Stenosis Classification and Diagnosis. Eur. J. Clin. Investig..

[88] Kalapos A., Szabó L., Dohy Z., Kiss M., Merkely B., Gyires-Tóth B., Vágó H. (2023). Automated T1 and T2 Mapping Segmentation on Cardiovascular Magnetic Resonance Imaging Using Deep Learning. Front. Cardiovasc. Med..

[89] Ishikita A., McIntosh C., Hanneman K., Lee M.M., Liang T., Karur G.R., Roche S.L., Hickey E., Geva T., Barron D.J. (2023). Machine Learning for Prediction of Adverse Cardiovascular Events in Adults with Repaired Tetralogy of Fallot Using Clinical and Cardiovascular Magnetic Resonance Imaging Variables. Circ. Cardiovasc. Imaging.

[90] De Vries I.R., van Laar J.O.E.H., van der Hout-van der Jagt M.B., Clur S.-A.B., Vullings R. (2023). Fetal Electrocardiography and Artificial Intelligence for Prenatal Detection of Congenital Heart Disease. Acta Obstet. Gynecol. Scand..

[91] Lv J., Dong B., Lei H., Shi G., Wang H., Zhu F., Wen C., Zhang Q., Fu L., Gu X. (2021). Artificial Intelligence-Assisted Auscultation in Detecting Congenital Heart Disease. Eur. Heart J. Digit. Health.

[92] Majeed A., Rofeberg V., Bellinger D.C., Wypij D., Newburger J.W. (2022). Machine Learning to Predict Executive Function in Adolescents with Repaired D-Transposition of the Great Arteries, Tetralogy of Fallot, and Fontan Palliation. J. Pediatr..

[93] Sakai A., Komatsu M., Komatsu R., Matsuoka R., Yasutomi S., Dozen A., Shozu K., Arakaki T., Machino H., Asada K. (2022). Medical Professional Enhancement Using Explainable Artificial Intelligence in Fetal Cardiac Ultrasound Screening. Biomedicines.

[94] Gearhart A., Goto S., Deo R.C., Powell A.J. (2022). An Automated View Classification Model for Pediatric Echocardiography Using Artificial Intelligence. J. Am. Soc. Echocardiogr..

[95] Valente Silva B., Marques J., Nobre Menezes M., Oliveira A.L., Pinto F.J. (2023). Artificial Intelligence-Based Diagnosis of Acute Pulmonary Embolism: Development of a Machine Learning Model Using 12-Lead Electrocardiogram. Rev. Port. Cardiol..

[96] Adedinsewo D., Hardway H.D., Morales-Lara A.C., Wieczorek M.A., Johnson P.W., Douglass E.J., Dangott B.J., Nakhleh R.E., Narula T., Patel P.C. (2023). Non-Invasive Detection of Cardiac Allograft Rejection among Heart Transplant Recipients Using an Electrocardiogram Based Deep Learning Model. Eur. Heart J. Digit. Health.

[97] Shiraishi Y., Goto S., Niimi N., Katsumata Y., Goda A., Takei M., Saji M., Sano M., Fukuda K., Kohno T. (2023). Improved Prediction of Sudden Cardiac Death in Patients with Heart Failure through Digital Processing of Electrocardiography. Europace.

[98] Hirota N., Suzuki S., Motogi J., Nakai H., Matsuzawa W., Takayanagi T., Umemoto T., Hyodo A., Satoh K., Arita T. (2023). Cardiovascular Events and Artificial Intelligence-Predicted Age Using 12-Lead Electrocardiograms. Int. J. Cardiol. Heart Vasc..

[99] Wouters P.C., van de Leur R.R., Vessies M.B., van Stipdonk A.M.W., Ghossein M.A., Hassink R.J., Doevendans P.A., van der Harst P., Maass A.H., Prinzen F.W. (2023). Electrocardiogram-Based Deep Learning Improves Outcome Prediction Following Cardiac Resynchronization Therapy. Eur. Heart J..

[100] Liu C.-W., Wu F.-H., Hu Y.-L., Pan R.-H., Lin C.-H., Chen Y.-F., Tseng G.-S., Chan Y.-K., Wang C.-L. (2023). Left Ventricular Hypertrophy Detection Using Electrocardiographic Signal. Sci. Rep..

[101] Zaver H.B., Mzaik O., Thomas J., Roopkumar J., Adedinsewo D., Keaveny A.P., Patel T. (2023). Utility of an Artificial Intelligence Enabled Electrocardiogram for Risk Assessment in Liver Transplant Candidates. Dig. Dis. Sci..

[102] Naser J.A., Lopez-Jimenez F., Chang A.Y., Baez-Suarez A., Attia Z.I., Pislaru S.V., Pellikka P.A., Lin G., Kapa S., Friedman P.A. (2023). Artificial Intelligence-Augmented Electrocardiogram in Determining Sex. Mayo Clin. Proc..

[103] Vaid A., Argulian E., Lerakis S., Beaulieu-Jones B.K., Krittanawong C., Klang E., Lampert J., Reddy V.Y., Narula J., Nadkarni G.N. (2023). Multi-Center Retrospective Cohort Study Applying Deep Learning to Electrocardiograms to Identify Left Heart Valvular Dysfunction. Commun. Med..

[104] Khan M.S., Arshad M.S., Greene S.J., Van Spall H.G.C., Pandey A., Vemulapalli S., Perakslis E., Butler J. (2023). Artificial Intelligence and Heart Failure: A State-of-the-art Review. Eur. J. Heart Fail..

[105] Almujally N.A., Aljrees T., Saidani O., Umer M., Faheem Z.B., Abuzinadah N., Alnowaiser K., Ashraf I. (2023). Monitoring Acute Heart Failure Patients Using Internet-of-Things-Based Smart Monitoring System. Sensors.

[106] Kobayashi M., Huttin O., Magnusson M., Ferreira J.P., Bozec E., Huby A.-C., Preud’homme G., Duarte K., Lamiral Z., Dalleau K. (2022). Machine Learning-Derived Echocardiographic Phenotypes Predict Heart Failure Incidence in Asymptomatic Individuals. JACC Cardiovasc. Imaging.

[107] Segar M.W., Patel K.V., Ayers C., Basit M., Tang W.H.W., Willett D., Berry J., Grodin J.L., Pandey A. (2020). Phenomapping of Patients with Heart Failure with Preserved Ejection Fraction Using Machine Learning-based Unsupervised Cluster Analysis. Eur. J. Heart Fail..

[108] Bourazana A., Xanthopoulos A., Briasoulis A., Magouliotis D., Spiliopoulos K., Athanasiou T., Vassilopoulos G., Skoularigis J., Triposkiadis F. (2024). Artificial Intelligence in Heart Failure: Friend or Foe?. Life.

[109] Bachtiger P., Petri C.F., Scott F.E., Ri Park S., Kelshiker M.A., Sahemey H.K., Dumea B., Alquero R., Padam P.S., Hatrick I.R. (2022). Point-of-Care Screening for Heart Failure with Reduced Ejection Fraction Using Artificial Intelligence during ECG-Enabled Stethoscope Examination in London, UK: A Prospective, Observational, Multicentre Study. Lancet Digit. Health.

[110] Harmon D.M., Witt D.R., Friedman P.A., Attia Z.I. (2021). Diagnosis and Treatment of New Heart Failure with Reduced Ejection Fraction by the Artificial Intelligence–Enhanced Electrocardiogram. Cardiovasc. Digit. Health J..

[111] Kwon J.-M., Kim K.-H., Eisen H.J., Cho Y., Jeon K.-H., Lee S.Y., Park J., Oh B.-H. (2021). Artificial Intelligence Assessment for Early Detection of Heart Failure with Preserved Ejection Fraction Based on Electrocardiographic Features. Eur. Heart J. Digit. Health.

[112] Wu J., Biswas D., Ryan M., Bernstein B.S., Rizvi M., Fairhurst N., Kaye G., Baral R., Searle T., Melikian N. (2024). Artificial Intelligence Methods for Improved Detection of Undiagnosed Heart Failure with Preserved Ejection Fraction. Eur. J. Heart Fail..

[113] Akerman A.P., Porumb M., Scott C.G., Beqiri A., Chartsias A., Ryu A.J., Hawkes W., Huntley G.D., Arystan A.Z., Kane G.C. (2023). Automated Echocardiographic Detection of Heart Failure with Preserved Ejection Fraction Using Artificial Intelligence. JACC Adv..

[114] Pană M.-A., Busnatu Ștefan S., Serbanoiu L.-I., Vasilescu E., Popescu N., Andrei C., Sinescu C.-J. (2021). Reducing the Heart Failure Burden in Romania by Predicting Congestive Heart Failure Using Artificial Intelligence: Proof of Concept. Appl. Sci..

[115] Cheungpasitporn W., Thongprayoon C., Kashani K.B. (2024). Artificial Intelligence in Heart Failure and Acute Kidney Injury: Emerging Concepts and Controversial Dimensions. Cardiorenal Med..

[116] Kamio T., Ikegami M., Machida Y., Uemura T., Chino N., Iwagami M. (2023). Machine Learning-Based Prognostic Modeling of Patients with Acute Heart Failure Receiving Furosemide in Intensive Care Units. Digit. Health.

[117] Naruka V., Arjomandi Rad A., Subbiah Ponniah H., Francis J., Vardanyan R., Tasoudis P., Magouliotis D.E., Lazopoulos G.L., Salmasi M.Y., Athanasiou T. (2022). Machine Learning and Artificial Intelligence in Cardiac Transplantation: A Systematic Review. Artif. Organs.

[118] Briasoulis A., Moustakidis S., Tzani A., Doulamis I., Kampaktsis P. (2021). Prediction of Outcomes after Heart Transplantation by Machine Learning Models. Eur. Heart J..

[119] Seraphin T.P., Luedde M., Roderburg C., van Treeck M., Scheider P., Buelow R.D., Boor P., Loosen S.H., Provaznik Z., Mendelsohn D. (2023). Prediction of Heart Transplant Rejection from Routine Pathology Slides with Self-Supervised Deep Learning. Eur. Heart J. Digit. Health.

[120] Ozcan I., Toya T., Cohen-Shelly M., Park H.W., Ahmad A., Ozcan A., Noseworthy P.A., Kapa S., Lerman L.O., Attia Z.I. (2022). Artificial Intelligence–Derived Cardiac Ageing Is Associated with Cardiac Events Post-Heart Transplantation. Eur. Heart J. Digit. Health.

[121] Sharma S., Menon N., Ruiz J., Luce C., Brumble L., Bhattacharya A., Goswami R. (2023). Developing a Risk Prediction Model for COVID-19 Infection in Heart Transplant Recipients Using Artificial Intelligence. Future Virol..

[122] Glass C., Davis R., Xiong B., Dov D., Glass M. (2020). The Use of Artificial Intelligence (AI) Machine Learning to Determine Myocyte Damage in Cardiac Transplant Acute Cellular Rejection. J. Heart Lung Transplant..

[123] Al-Ani M.A., Bai C., Shickel B., Bledsoe M., Ahmed M.M., Vilaro J., Parker A., Aranda J., Jeng E., Bleiweis M. (2023). (750) Determinants of Successful Bridging to Heart Transplantation on Temporary Percutaneous Left Ventricular Support—An Insight Using Artificial Intelligence. J. Heart Lung Transplant..

[124] Peyster E.G., Arabyarmohammadi S., Janowczyk A., Azarianpour-Esfahani S., Sekulic M., Cassol C., Blower L., Parwani A., Lal P., Feldman M.D. (2021). An Automated Computational Image Analysis Pipeline for Histological Grading of Cardiac Allograft Rejection. Eur. Heart J..

[125] Lipkova J., Chen T.Y., Lu M.Y., Chen R.J., Shady M., Williams M., Wang J., Noor Z., Mitchell R.N., Turan M. (2022). Deep Learning-Enabled Assessment of Cardiac Allograft Rejection from Endomyocardial Biopsies. Nat. Med..

[126] Giuste F.O., Sequeira R., Keerthipati V., Lais P., Mirzazadeh A., Mohseni A., Zhu Y., Shi W., Marteau B., Zhong Y. (2023). Explainable Synthetic Image Generation to Improve Risk Assessment of Rare Pediatric Heart Transplant Rejection. J. Biomed. Inform..

[127] Lisboa P.J.G., Jayabalan M., Ortega-Martorell S., Olier I., Medved D., Nilsson J. (2022). Enhanced Survival Prediction Using Explainable Artificial Intelligence in Heart Transplantation. Sci. Rep..

[128] Ruiz Morales J., Nativi-Nicolau J., Jang J., Patel P., Yip D., Leoni-Moreno J., Goswami R. (2022). Artificial Intelligence 12 Lead ECG Based Heart Age Estimation and 1-Year Outcomes after Heart Transplantation. J. Heart Lung Transplant..

[129] Agasthi P., Smith S.D., Murphy K.M., Buras M.R., Golafshar M., Herner M., Anand S., Pujari S., Allam M.N., Mookadam F. (2020). Artificial Intelligence Helps Predict 5-Year Mortality and Graft Failure in Patients Undergoing Orthotopic Heart Transplantation. J. Heart Lung Transplant..

[130] Ozcan I., Toya T., Cohen-Shelly M., Ahmad A., Corban M.T., Noseworthy P.A., Kapa S., Lerman L.O., Attia Z.I., Friedman P.A. (2021). Artificial Intelligence Derived Age Algorithm after Heart Transplantation. Eur. Heart J..

[131] Soh D.C.K., Ng E.Y.K., Jahmunah V., Oh S.L., San T.R., Acharya U.R. (2020). A Computational Intelligence Tool for the Detection of Hypertension Using Empirical Mode Decomposition. Comput. Biol. Med..

[132] López-Martínez F., Núñez-Valdez E.R., Crespo R.G., García-Díaz V. (2020). An Artificial Neural Network Approach for Predicting Hypertension Using NHANES Data. Sci. Rep..

[133] Wu X., Yuan X., Wang W., Liu K., Qin Y., Sun X., Ma W., Zou Y., Zhang H., Zhou X. (2020). Value of a Machine Learning Approach for Predicting Clinical Outcomes in Young Patients with Hypertension. Hypertension.

[134] Aziz F., Malek S., Mhd Ali A., Wong M.S., Mosleh M., Milow P. (2020). Determining Hypertensive Patients’ Beliefs towards Medication and Associations with Medication Adherence Using Machine Learning Methods. PeerJ.

[135] Koshimizu H., Kojima R., Kario K., Okuno Y. (2020). Prediction of Blood Pressure Variability Using Deep Neural Networks. Int. J. Med. Inform..

[136] Hamoud B., Kashevnik A., Othman W., Shilov N. (2023). Neural Network Model Combination for Video-Based Blood Pressure Estimation: New Approach and Evaluation. Sensors.

[137] Cheng H., Xiong J., Chen Z., Chen J. (2023). Deep Learning-Based Non-Contact IPPG Signal Blood Pressure Measurement Research. Sensors.

[138] Xing W., Shi Y., Wu C., Wang Y., Wang X. (2023). Predicting Blood Pressure from Face Videos Using Face Diagnosis Theory and Deep Neural Networks Technique. Comput. Biol. Med..

[139] Visco V., Izzo C., Mancusi C., Rispoli A., Tedeschi M., Virtuoso N., Giano A., Gioia R., Melfi A., Serio B. (2023). Artificial Intelligence in Hypertension Management: An Ace up Your Sleeve. J. Cardiovasc. Dev. Dis..

[140] Maqsood S., Xu S., Tran S., Garg S., Springer M., Karunanithi M., Mohawesh R. (2022). A Survey: From Shallow to Deep Machine Learning Approaches for Blood Pressure Estimation Using Biosensors. Expert Syst. Appl..

[141] Herzog L., Ilan Ber R., Horowitz-Kugler Z., Rabi Y., Brufman I., Paz Y.E., Lopez-Jimenez F. (2023). Causal Deep Neural Network-Based Model for First-Line Hypertension Management. Mayo Clin. Proc. Digit. Health.

[142] Khthir R., Santhanam P. (2021). Artificial Intelligence (AI) Approach to Identifying Factors That Determine Systolic Blood Pressure in Type 2 Diabetes (Study from the LOOK AHEAD Cohort). Diabetes Metab. Syndr..

[143] Aryal S., Manandhar I., Mei X., Yeoh B.S., Tummala R., Saha P., Osman I., Zubcevic J., Durgan D.J., Vijay-Kumar M. (2023). Combating Hypertension beyond GWAS: Microbiome and Artificial Intelligence as Opportunities for Precision Medicine. Camb. Prisms Precis. Med..

[144] Lin Z., Cheng Y.T., Cheung B.M.Y. (2023). Machine Learning Algorithms Identify Hypokalaemia Risk in People with Hypertension in the United States National Health and Nutrition Examination Survey 1999–2018. Ann. Med..

[145] Kusunose K., Hirata Y., Tsuji T., Kotoku J., Sata M. (2020). Deep Learning to Predict Elevated Pulmonary Artery Pressure in Patients with Suspected Pulmonary Hypertension Using Standard Chest X ray. Sci. Rep..

[146] Hardacre C.J., Robertshaw J.A., Barratt S.L., Adams H.L., MacKenzie Ross R.V., Robinson G.R.E., Suntharalingam J., Pauling J.D., Rodrigues J.C.L. (2021). Diagnostic Test Accuracy of Artificial Intelligence Analysis of Cross-Sectional Imaging in Pulmonary Hypertension: A Systematic Literature Review. Br. J. Radiol..

[147] Ragnarsdottir H., Manduchi L., Michel H., Laumer F., Wellmann S., Ozkan E., Vogt J.E. (2022). Interpretable Prediction of Pulmonary Hypertension in Newborns Using Echocardiograms. Lecture Notes in Computer Science.

[148] Chakravarty K., Antontsev V.G., Khotimchenko M., Gupta N., Jagarapu A., Bundey Y., Hou H., Maharao N., Varshney J. (2021). Accelerated Repurposing and Drug Development of Pulmonary Hypertension Therapies for COVID-19 Treatment Using an AI-Integrated Biosimulation Platform. Molecules.

[149] Rahaghi F.N., Nardelli P., Harder E., Singh I., Sánchez-Ferrero G.V., Ross J.C., San José Estépar R., Ash S.Y., Hunsaker A.R., Maron B.A. (2021). Quantification of Arterial and Venous Morphologic Markers in Pulmonary Arterial Hypertension Using CT Imaging. Chest.

[150] Shi B., Zhou T., Lv S., Wang M., Chen S., Heidari A.A., Huang X., Chen H., Wang L., Wu P. (2022). An Evolutionary Machine Learning for Pulmonary Hypertension Animal Model from Arterial Blood Gas Analysis. Comput. Biol. Med..

[151] Amodeo I., De Nunzio G., Raffaeli G., Borzani I., Griggio A., Conte L., Macchini F., Condò V., Persico N., Fabietti I. (2021). A maChine and Deep Learning Approach to Predict pulmoNary hyperteNsIon in newbornS with Congenital Diaphragmatic Hernia (CLANNISH): Protocol for a Retrospective Study. PLoS ONE.

[152] Van der Bijl P., Bax J.J. (2022). Using Deep Learning to Diagnose Pulmonary Hypertension. Eur. Heart J. Cardiovasc. Imaging.

[153] Swift A.J., Lu H., Uthoff J., Garg P., Cogliano M., Taylor J., Metherall P., Zhou S., Johns C.S., Alabed S. (2021). A Machine Learning Cardiac Magnetic Resonance Approach to Extract Disease Features and Automate Pulmonary Arterial Hypertension Diagnosis. Eur. Heart J. Cardiovasc. Imaging.

[154] Charters P.F.P., Rossdale J., Brown W., Burnett T.A., Komber H.M.E.I., Thompson C., Robinson G., MacKenzie Ross R., Suntharalingam J., Rodrigues J.C.L. (2022). Diagnostic Accuracy of an Automated Artificial Intelligence Derived Right Ventricular to Left Ventricular Diameter Ratio Tool on CT Pulmonary Angiography to Predict Pulmonary Hypertension at Right Heart Catheterisation. Clin. Radiol..

[155] Fortmeier V., Lachmann M., Körber M.I., Unterhuber M., von Scheidt M., Rippen E., Harmsen G., Gerçek M., Friedrichs K.P., Roder F. (2022). Solving the Pulmonary Hypertension Paradox in Patients with Severe Tricuspid Regurgitation by Employing Artificial Intelligence. JACC Cardiovasc. Interv..

[156] Liu C.-M., Shih E.S.C., Chen J.-Y., Huang C.-H., Wu I.-C., Chen P.-F., Higa S., Yagi N., Hu Y.-F., Hwang M.-J. (2022). Artificial Intelligence-Enabled Electrocardiogram Improves the Diagnosis and Prediction of Mortality in Patients with Pulmonary Hypertension. JACC Asia.

[157] Lu W., Huang J., Shen Q., Sun F., Li J. (2023). Identification of Diagnostic Biomarkers for Idiopathic Pulmonary Hypertension with Metabolic Syndrome by Bioinformatics and Machine Learning. Sci. Rep..

[158] Yu X., Qin W., Lin X., Shan Z., Huang L., Shao Q., Wang L., Chen M. (2023). Synergizing the Enhanced RIME with Fuzzy K-Nearest Neighbor for Diagnose of Pulmonary Hypertension. Comput. Biol. Med..

[159] Hyde B., Paoli C.J., Panjabi S., Bettencourt K.C., Bell Lynum K.S., Selej M. (2023). A Claims-based, Machine-learning Algorithm to Identify Patients with Pulmonary Arterial Hypertension. Pulm. Circ..

[160] Zhang N., Zhao X., Li J., Huang L., Li H., Feng H., Garcia M.A., Cao Y., Sun Z., Chai S. (2023). Machine Learning Based on Computed Tomography Pulmonary Angiography in Evaluating Pulmonary Artery Pressure in Patients with Pulmonary Hypertension. J. Clin. Med..

[161] Hirata Y., Tsuji T., Kotoku J., Sata M., Kusunose K. (2024). Echocardiographic Artificial Intelligence for Pulmonary Hypertension Classification. Heart.

[162] Imai S., Sakao S., Nagata J., Naito A., Sekine A., Sugiura T., Shigeta A., Nishiyama A., Yokota H., Shimizu N. (2024). Artificial Intelligence-Based Model for Predicting Pulmonary Arterial Hypertension on Chest X-ray Images. BMC Pulm. Med..

[163] Ragnarsdottir H., Ozkan E., Michel H., Chin-Cheong K., Manduchi L., Wellmann S., Vogt J.E. (2024). Deep Learning Based Prediction of Pulmonary Hypertension in Newborns Using Echocardiograms. Int. J. Comput. Vis..

[164] Dwivedi K., Sharkey M., Delaney L., Alabed S., Rajaram S., Hill C., Johns C., Rothman A., Mamalakis M., Thompson A.A.R. (2024). Improving Prognostication in Pulmonary Hypertension Using AI-Quantified Fibrosis and Radiologic Severity Scoring at Baseline CT. Radiology.

[165] Griffiths M., Manlhiot C., Chinni B.K., Sleeper L.A., Abman S., Rosenzweig E., Romer L.H., Mullen M.P., Lin S., Benza R. (2023). Abstract 15889: An Artificial Intelligence-Derived Pediatric Pulmonary Hypertension Risk Prediction Model from the Pediatric Pulmonary Hypertension Network (PPHNet) Registry. Circulation.

[166] Mamalakis M., Dwivedi K., Sharkey M., Alabed S., Kiely D., Swift A.J. (2023). A Transparent Artificial Intelligence Framework to Assess Lung Disease in Pulmonary Hypertension. Sci. Rep..

[167] Tchuente Foguem G., Coulibaly L., Diamoutene A. (2024). Combined Learning Models for Survival Analysis of Patients with Pulmonary Hypertension. Intell. Syst. Appl..

[168] Han P.-L., Jiang L., Cheng J.-L., Shi K., Huang S., Jiang Y., Jiang L., Xia Q., Li Y.-Y., Zhu M. (2024). Artificial Intelligence-Assisted Diagnosis of Congenital Heart Disease and Associated Pulmonary Arterial Hypertension from Chest Radiographs: A Multi-Reader Multi-Case Study. Eur. J. Radiol..

[169] Anand V., Weston A.D., Scott C.G., Kane G.C., Pellikka P.A., Carter R.E. (2024). Machine Learning for Diagnosis of Pulmonary Hypertension by Echocardiography. Mayo Clin. Proc..

[170] Lai C.K.-C., Leung E., He Y., Cheung C.-C., Oliver M.O.Y., Yu Q., Li T.C.-M., Lee A.L.-H., Yu L., Lui G.C.-Y. (2024). A Machine Learning-Based Risk Score for Prediction of Infective Endocarditis among Patients with *Staphylococcus Aureus* Bacteraemia—The SABIER Score. J. Infect. Dis..

[171] Yi C., Zhang H., Yang J., Chen D., Jiang S. (2024). Elucidating Common Pathogenic Transcriptional Networks in Infective Endocarditis and Sepsis: Integrated Insights from Biomarker Discovery and Single-Cell RNA Sequencing. Front. Immunol..

[172] Galizzi Fae I., Murta Pinto P.H.O., De Oliveira G.B., Taconeli C.A., De Andrade A.B., De Padua L.B., Diamante L.C., Ferrari T.C.A., Nunes M.C.P. (2023). Cardiac Complications as a Major Predictor of In-Hospital Death in Infective Endocarditis Using Machine-Learning Algorithm Analysis. Eur. Heart J..

[173] Chen Z., Lalande A., Salomon M., Decourselle T., Pommier T., Qayyum A., Shi J., Perrot G., Couturier R. (2022). Automatic Deep Learning-Based Myocardial Infarction Segmentation from Delayed Enhancement MRI. Comput. Med. Imaging Graph..

[174] Rauseo E., Izquierdo Morcillo C., Raisi-Estabragh Z., Gkontra P., Aung N., Lekadir K., Petersen S.E. (2021). New Imaging Signatures of Cardiac Alterations in Ischaemic Heart Disease and Cerebrovascular Disease Using CMR Radiomics. Front. Cardiovasc. Med..

[175] Liu W.-C., Lin C., Lin C.-S., Tsai M.-C., Chen S.-J., Tsai S.-H., Lin W.-S., Lee C.-C., Tsao T.-P., Cheng C.-C. (2021). An Artificial Intelligence-Based Alarm Strategy Facilitates Management of Acute Myocardial Infarction. J. Pers. Med..

[176] Zhao Y., Xiong J., Hou Y., Zhu M., Lu Y., Xu Y., Teliewubai J., Liu W., Xu X., Li X. (2020). Early Detection of ST-Segment Elevated Myocardial Infarction by Artificial Intelligence with 12-Lead Electrocardiogram. Int. J. Cardiol..

[177] Cho Y., Kwon J.-M., Kim K.-H., Medina-Inojosa J.R., Jeon K.-H., Cho S., Lee S.Y., Park J., Oh B.-H. (2020). Artificial Intelligence Algorithm for Detecting Myocardial Infarction Using Six-Lead Electrocardiography. Sci. Rep..

[178] Liu W.-C., Lin C.-S., Tsai C.-S., Tsao T.-P., Cheng C.-C., Liou J.-T., Lin W.-S., Cheng S.-M., Lou Y.-S., Lee C.-C. (2021). A Deep Learning Algorithm for Detecting Acute Myocardial Infarction. EuroIntervention.

[179] Velusamy D., Ramasamy K. (2021). Ensemble of Heterogeneous Classifiers for Diagnosis and Prediction of Coronary Artery Disease with Reduced Feature Subset. Comput. Methods Programs Biomed..

[180] Muhammad L.J., Al-Shourbaji I., Haruna A.A., Mohammed I.A., Ahmad A., Jibrin M.B. (2021). Machine Learning Predictive Models for Coronary Artery Disease. SN Comput. Sci..

[181] Li D., Xiong G., Zeng H., Zhou Q., Jiang J., Guo X. (2021). Machine Learning-Aided Risk Stratification System for the Prediction of Coronary Artery Disease. Int. J. Cardiol..

[182] Brendel J.M., Walterspiel J., Hagen F., Kübler J., Paul J.-F., Nikolaou K., Gawaz M., Greulich S., Krumm P., Winkelmann M. (2024). Coronary Artery Disease Evaluation during Transcatheter Aortic Valve Replacement Work-up Using Photon-Counting CT and Artificial Intelligence. Diagn. Interv. Imaging.

[183] Ihdayhid A.R., Sehly A., He A., Joyner J., Flack J., Konstantopoulos J., Newby D.E., Williams M.C., Ko B.S., Chow B.J.W. (2024). Coronary Artery Stenosis and High-Risk Plaque Assessed with an Unsupervised Fully Automated Deep Learning Technique. JACC Adv..

[184] Uzokov J., Alyavi A., Alyavi B., Abdullaev A. (2024). How Artificial Intelligence Can Assist with Ischaemic Heart Disease. Eur. Heart J..

[185] Abdelrahman K., Shiyovich A., Huck D., Berman A., Weber B., Gupta S., Cardoso R., Blankstein R. (2024). Artificial Intelligence in Coronary Artery Calcium Scoring Detection and Quantification. Diagnostics.

[186] Park M.J., Choi Y.J., Shim M., Cho Y., Park J., Choi J., Kim J., Lee E., Kim S.-Y. (2024). Performance of ECG-Derived Digital Biomarker for Screening Coronary Occlusion in Resuscitated out-of-Hospital Cardiac Arrest Patients: A Comparative Study between Artificial Intelligence and a Group of Experts. J. Clin. Med..

[187] Alkhamis M.A., Al Jarallah M., Attur S., Zubaid M. (2024). Interpretable Machine Learning Models for Predicting In-Hospital and 30 Days Adverse Events in Acute Coronary Syndrome Patients in Kuwait. Sci. Rep..

[188] Zhu X., Xie B., Chen Y., Zeng H., Hu J. (2024). Machine Learning in the Prediction of In-Hospital Mortality in Patients with First Acute Myocardial Infarction. Clin. Chim. Acta.

[189] Kasim S., Amir Rudin P.N.F., Malek S., Aziz F., Wan Ahmad W.A., Ibrahim K.S., Muhmad Hamidi M.H., Raja Shariff R.E., Fong A.Y.Y., Song C. (2024). Data Analytics Approach for Short- and Long-Term Mortality Prediction Following Acute Non-ST-Elevation Myocardial Infarction (NSTEMI) and Unstable Angina (UA) in Asians. PLoS ONE.

[190] Oliveira M., Seringa J., Pinto F.J., Henriques R., Magalhães T. (2023). Machine Learning Prediction of Mortality in Acute Myocardial Infarction. BMC Med. Inform. Decis. Mak..

[191] Azdaki N., Salmani F., Kazemi T., Partovi N., Bizhaem S.K., Moghadam M.N., Moniri Y., Zarepur E., Mohammadifard N., Alikhasi H. (2024). Which Risk Factor Best Predicts Coronary Artery Disease Using Artificial Neural Network Method?. BMC Med. Inform. Decis. Mak..

[192] Zhan W., Hu H., Hao B., Zhu H., Yan T., Zhang J., Wang S., Liu S., Zhang T. (2024). Development of Machine Learning-Based Malignant Pericardial Effusion-Related Model in Breast Cancer: Implications for Clinical Significance, Tumor Immune and Drug-Therapy. Heliyon.

[193] Liu Y.-L., Lin C.-S., Cheng C.-C., Lin C. (2022). A Deep Learning Algorithm for Detecting Acute Pericarditis by Electrocardiogram. J. Pers. Med..

[194] Cheng C.-Y., Wu C.-C., Chen H.-C., Hung C.-H., Chen T.-Y., Lin C.-H.R., Chiu I.-M. (2023). Development and Validation of a Deep Learning Pipeline to Measure Pericardial Effusion in Echocardiography. Front. Cardiovasc. Med..

[195] Wilder-Smith A.J., Yang S., Weikert T., Bremerich J., Haaf P., Segeroth M., Ebert L.C., Sauter A., Sexauer R. (2022). Automated Detection, Segmentation, and Classification of Pericardial Effusions on Chest CT Using a Deep Convolutional Neural Network. Diagnostics.

[196] Piccini J.P., Cunnane R., Steffel J., El-Chami M.F., Reynolds D., Roberts P.R., Soejima K., Steinwender C., Garweg C., Chinitz L. (2022). Development and Validation of a Risk Score for Predicting Pericardial Effusion in Patients Undergoing Leadless Pacemaker Implantation: Experience with the Micra Transcatheter Pacemaker. Europace.

[197] McBane R.D., Murphree D.H., Liedl D., Lopez-Jimenez F., Attia I.Z., Arruda-Olson A.M., Scott C.G., Prodduturi N., Nowakowski S.E., Rooke T.W. (2024). Artificial Intelligence of Arterial Doppler Waveforms to Predict Major Adverse Outcomes among Patients Evaluated for Peripheral Artery Disease. J. Am. Heart Assoc..

[198] Rusinovich Y., Rusinovich V., Buhayenka A., Liashko V., Sabanov A., Holstein D.J.F., Aldmour S., Doss M., Branzan D. (2024). Classification of Anatomic Patterns of Peripheral Artery Disease with Automated Machine Learning (AutoML). Vascular.

[199] Sasikala P., Mohanarathinam A. (2024). A Powerful Peripheral Arterial Disease Detection Using Machine Learning-Based Severity Level Classification Model and Hyper Parameter Optimization Methods. Biomed. Signal Process. Control.

[200] Li B., Shaikh F., Zamzam A., Syed M.H., Abdin R., Qadura M. (2024). A Machine Learning Algorithm for Peripheral Artery Disease Prognosis Using Biomarker Data. iScience.

[201] Masoumi Shahrbabak S., Kim S., Youn B.D., Cheng H.-M., Chen C.-H., Mukkamala R., Hahn J.-O. (2024). Peripheral Artery Disease Diagnosis Based on Deep Learning-Enabled Analysis of Non-Invasive Arterial Pulse Waveforms. Comput. Biol. Med..

[202] McBane R.D., Murphree D.H., Liedl D., Lopez-Jimenez F., Arruda-Olson A., Scott C.G., Prodduturi N., Nowakowski S.E., Rooke T.W., Casanegra A.I. (2024). Artificial Intelligence of Arterial Doppler Waveforms to Predict Major Adverse Outcomes among Patients with Diabetes Mellitus. J. Vasc. Surg..

[203] Li B., Eisenberg N., Beaton D., Lee D.S., Aljabri B., Verma R., Wijeysundera D.N., Rotstein O.D., de Mestral C., Mamdani M. (2024). Using Machine Learning (XGBoost) to Predict Outcomes after Infrainguinal Bypass for Peripheral Artery Disease. Ann. Surg..

[204] Liu L., Bi B., Cao L., Gui M., Ju F. (2024). Predictive Model, and Risk Analysis for Peripheral Vascular Disease in Type 2 Diabetes Mellitus Patients Using Machine Learning and Shapley Additive Explanation. Front. Endocrinol..

[205] Nassour N., Akhbari B., Ranganathan N., Shin D., Ghaednia H., Ashkani-Esfahani S., DiGiovanni C.W., Guss D. (2024). Using Machine Learning in the Prediction of Symptomatic Venous Thromboembolism Following Ankle Fracture. Foot Ankle Surg..

[206] Chen R., Petrazzini B.O., Malick W.A., Rosenson R.S., Do R. (2024). Prediction of Venous Thromboembolism in Diverse Populations Using Machine Learning and Structured Electronic Health Records. Arterioscler. Thromb. Vasc. Biol..

[207] Pan S., Bian L., Luo H., Conway A., Qiao W., Win T., Wang W. (2024). Risk Factor Analysis and Prediction Model Construction for Surgical Patients with Venous Thromboembolism: A Prospective Study. Interdiscip. Nurs. Res..

[208] Grdinic A.G., Radovanovic S., Gleditsch J., Jørgensen C.T., Asady E., Pettersen H.H., Delibasic B., Ghanima W. (2024). Developing a Machine Learning Model for Bleeding Prediction in Patients with Cancer-Associated Thrombosis Receiving Anticoagulation Therapy. J. Thromb. Haemost..

[209] Chiasakul T., Lam B.D., McNichol M., Robertson W., Rosovsky R.P., Lake L., Vlachos I.S., Adamski A., Reyes N., Abe K. (2023). Artificial Intelligence in the Prediction of Venous Thromboembolism: A Systematic Review and Pooled Analysis. Eur. J. Haematol..

[210] Wang X., Xi H., Geng X., Li Y., Zhao M., Li F., Li Z., Ji H., Tian H. (2023). Artificial Intelligence-Based Prediction of Lower Extremity Deep Vein Thrombosis Risk after Knee/Hip Arthroplasty. Clin. Appl. Thromb. Hemost..

[211] Wang K.Y., Ikwuezunma I., Puvanesarajah V., Babu J., Margalit A., Raad M., Jain A. (2023). Using Predictive Modeling and Supervised Machine Learning to Identify Patients at Risk for Venous Thromboembolism Following Posterior Lumbar Fusion. Glob. Spine J..

[212] Muñoz A.J., Souto J.C., Lecumberri R., Obispo B., Sanchez A., Aparicio J., Aguayo C., Gutierrez D., Palomo A.G., Fanjul V. (2023). Development of a Predictive Model of Venous Thromboembolism Recurrence in Anticoagulated Cancer Patients Using Machine Learning. Thromb. Res..

[213] Razzaq M., Goumidi L., Iglesias M.-J., Munsch G., Bruzelius M., Ibrahim-Kosta M., Butler L., Odeberg J., Morange P.-E., Tregouet D.A. (2021). Explainable Artificial Neural Network for Recurrent Venous Thromboembolism Based on Plasma Proteomics. Computational Methods in Systems Biology.

[214] Contreras-Luján E.E., García-Guerrero E.E., López-Bonilla O.R., Tlelo-Cuautle E., López-Mancilla D., Inzunza-González E. (2022). Evaluation of Machine Learning Algorithms for Early Diagnosis of Deep Venous Thrombosis. Math. Comput. Appl..

[215] Seo J.W., Park S., Kim Y.J., Hwang J.H., Yu S.H., Kim J.H., Kim K.G. (2023). Artificial Intelligence-Based Iliofemoral Deep Venous Thrombosis Detection Using a Clinical Approach. Sci. Rep..

[216] Alhwiti T., Aldrugh S., Megahed F.M. (2023). Predicting In-Hospital Mortality after Transcatheter Aortic Valve Replacement Using Administrative Data and Machine Learning. Sci. Rep..

[217] Strange G., Stewart S., Watts A., Playford D. (2023). Enhanced Detection of Severe Aortic Stenosis via Artificial Intelligence: A Clinical Cohort Study. Open Heart.

[218] Ueda D., Matsumoto T., Ehara S., Yamamoto A., Walston S.L., Ito A., Shimono T., Shiba M., Takeshita T., Fukuda D. (2023). Artificial Intelligence-Based Model to Classify Cardiac Functions from Chest Radiographs: A Multi-Institutional, Retrospective Model Development and Validation Study. Lancet Digit. Health.

[219] Singh S., Chaudhary R., Bliden K.P., Tantry U.S., Gurbel P.A., Visweswaran S., Harinstein M.E. (2024). Meta-Analysis of the Performance of AI-Driven ECG Interpretation in the Diagnosis of Valvular Heart Diseases. Am. J. Cardiol..

[220] Brown K., Roshanitabrizi P., Rwebembera J., Okello E., Beaton A., Linguraru M.G., Sable C.A. (2024). Using Artificial Intelligence for Rheumatic Heart Disease Detection by Echocardiography: Focus on Mitral Regurgitation. J. Am. Heart Assoc..

[221] Toggweiler S., Wyler von Ballmoos M.C., Moccetti F., Douverny A., Wolfrum M., Imamoglu Z., Mohler A., Gülan U., Kim W.-K. (2024). A Fully Automated Artificial Intelligence-Driven Software for Planning of Transcatheter Aortic Valve Replacement. Cardiovasc. Revasc. Med..

[222] Solomon M.D., Tabada G., Allen A., Sung S.H., Go A.S. (2021). Large-Scale Identification of Aortic Stenosis and Its Severity Using Natural Language Processing on Electronic Health Records. Cardiovasc. Digit. Health J..

[223] Aoyama G., Zhao L., Zhao S., Xue X., Zhong Y., Yamauchi H., Tsukihara H., Maeda E., Ino K., Tomii N. (2022). Automatic Aortic Valve Cusps Segmentation from CT Images Based on the Cascading Multiple Deep Neural Networks. J. Imaging.

[224] Dasi A., Lee B., Polsani V., Yadav P., Dasi L.P., Thourani V.H. (2024). Predicting Pressure Gradient Using Artificial Intelligence for Transcatheter Aortic Valve Replacement. JTCVS Technol..

[225] Krishna H., Desai K., Slostad B., Bhayani S., Arnold J.H., Ouwerkerk W., Hummel Y., Lam C.S.P., Ezekowitz J., Frost M. (2023). Fully Automated Artificial Intelligence Assessment of Aortic Stenosis by Echocardiography. J. Am. Soc. Echocardiogr..

[226] Xie L.-F., Xie Y.-L., Wu Q.-S., He J., Lin X.-F., Qiu Z.-H., Chen L.-W. (2024). A Predictive Model for Postoperative Adverse Outcomes Following Surgical Treatment of Acute Type A Aortic Dissection Based on Machine Learning. J. Clin. Hypertens..

[227] Zhou M., Lei L., Chen W., Luo Q., Li J., Zhou F., Yang X., Pan Y. (2024). Deep Learning-Based Diagnosis of Aortic Dissection Using an Electrocardiogram: Development, Validation, and Clinical Implications of the AADE Score. Kardiol. Pol..

[228] Irtyuga O., Babakekhyan M., Kostareva A., Uspensky V., Gordeev M., Faggian G., Malashicheva A., Metsker O., Shlyakhto E., Kopanitsa G. (2023). Analysis of Prevalence and Clinical Features of Aortic Stenosis in Patients with and without Bicuspid Aortic Valve Using Machine Learning Methods. J. Pers. Med..

[229] Kennedy L., Bates K., Therrien J., Grossman Y., Kodaira M., Pressacco J., Rosati A., Dagenais F., Leask R.L., Lachapelle K. (2023). Thoracic Aortic Aneurysm Risk Assessment. JACC Adv..

[230] Benjamin E.J., Muntner P., Alonso A., Bittencourt M.S., Callaway C.W., Carson A.P., Chamberlain A.M., Chang A.R., Cheng S., Das S.R. (2019). Heart Disease and Stroke Statistics—2019 Update: A Report from the American Heart Association. Circulation.

[231] Frohnert P.P., Gluliani E.R., Friedberg M., Johnson W.J., Tauxe W.N. (1970). Statistical Investigation of Correlations between Serum Potassium Levels and Electrocardiographic Findings in Patients on Intermittent Hemodialysis Therapy. Circulation.

[232] Martínez-Sellés M., Marina-Breysse M. (2023). Current and Future Use of Artificial Intelligence in Electrocardiography. J. Cardiovasc. Dev. Dis..

[233] Zhang Y., Xu S., Xing W., Chen Q., Liu X., Pu Y., Xin F., Jiang H., Yin Z., Tao D. (2024). Robust Artificial Intelligence Tool for Atrial Fibrillation Diagnosis: Novel Development Approach Incorporating Both Atrial Electrograms and Surface ECG and Evaluation by Head-to-head Comparison with Hospital-based Physician ECG Readers. J. Am. Heart Assoc..

[234] Kawamura Y., Vafaei Sadr A., Abedi V., Zand R. (2024). Many Models, Little Adoption—What Accounts for Low Uptake of Machine Learning Models for Atrial Fibrillation Prediction and Detection?. J. Clin. Med..

[235] Xie C., Wang Z., Yang C., Liu J., Liang H. (2024). Machine Learning for Detecting Atrial Fibrillation from ECGs: Systematic Review and Meta-Analysis. Rev. Cardiovasc. Med..

[236] Tehrani B.N., Truesdell A.G., Psotka M.A., Rosner C., Singh R., Sinha S.S., Damluji A.A., Batchelor W.B. (2020). A Standardized and Comprehensive Approach to the Management of Cardiogenic Shock. JACC Heart Fail..

[237] Raheem A., Waheed S., Karim M., Khan N.U., Jawed R. (2024). Prediction of Major Adverse Cardiac Events in the Emergency Department Using an Artificial Neural Network with a Systematic Grid Search. Int. J. Emerg. Med..

[238] Abusnina W., Elhouderi E., Walters R.W., Al-Abdouh A., Mostafa M.R., Liu J.L., Mazozy R., Mhanna M., Ben-Dor I., Dufani J. (2024). Sex Differences in the Clinical Outcomes of Patients with Takotsubo Stress Cardiomyopathy: A Meta-Analysis of Observational Studies. Am. J. Cardiol..

[239] Matta A., Delmas C., Campelo-Parada F., Lhermusier T., Bouisset F., Elbaz M., Nader V., Blanco S., Roncalli J., Carrié D. (2022). Takotsubo Cardiomyopathy. Rev. Cardiovasc. Med..

[240] Moynihan D., Monaco S., Ting T.W., Narasimhalu K., Hsieh J., Kam S., Lim J.Y., Lim W.K., Davila S., Bylstra Y. (2024). Cluster Analysis and Visualisation of Electronic Health Records Data to Identify Undiagnosed Patients with Rare Genetic Diseases. Sci. Rep..

[241] van Assen M., Razavi A.C., Whelton S.P., De Cecco C.N. (2023). Artificial Intelligence in Cardiac Imaging: Where We Are and What We Want. Eur. Heart J..

[242] Wehbe R.M., Katsaggelos A.K., Hammond K.J., Hong H., Ahmad F.S., Ouyang D., Shah S.J., McCarthy P.M., Thomas J.D. (2023). Deep Learning for Cardiovascular Imaging: A Review. JAMA Cardiol..

[243] Jone P.-N., Gearhart A., Lei H., Xing F., Nahar J., Lopez-Jimenez F., Diller G.-P., Marelli A., Wilson L., Saidi A. (2022). Artificial Intelligence in Congenital Heart Disease. JACC Adv..

[244] Dahiya E.S., Kalra A.M., Lowe A., Anand G. (2024). Wearable Technology for Monitoring Electrocardiograms (ECGs) in Adults: A Scoping Review. Sensors.

[245] Yoon M., Park J.J., Hur T., Hua C.-H., Hussain M., Lee S., Choi D.-J. (2024). Application and Potential of Artificial Intelligence in Heart Failure: Past, Present, and Future. Int. J. Heart Fail..

[246] Dogan S., Barua P.D., Tuncer T., Acharya U.R. (2024). An Accurate Hypertension Detection Model Based on a New Odd-Even Pattern Using Ballistocardiograph Signals. Eng. Appl. Artif. Intell..

[247] Becerra-Muñoz V.M., Gómez Sáenz J.T., Escribano Subías P. (2024). La importancia de los datos en la hipertensión arterial pulmonar: De los registros internacionales al machine learning. Med. Clin..

[248] Perek S., Nussinovitch U., Sagi N., Gidron Y., Raz-Pasteur A. (2023). Prognostic Implications of Ultra-Short Heart Rate Variability Indices in Hospitalized Patients with Infective Endocarditis. PLoS ONE.

[249] Virani S.S., Alonso A., Benjamin E.J., Bittencourt M.S., Callaway C.W., Carson A.P., Chamberlain A.M., Chang A.R., Cheng S., Delling F.N. (2020). Heart Disease and Stroke Statistics—2020 Update: A Report from the American Heart Association. Circulation.

[250] Uzun Ozsahin D., Ozgocmen C., Balcioglu O., Ozsahin I., Uzun B. (2022). Diagnostic AI and Cardiac Diseases. Diagnostics.

[251] El Sherbini A., Rosenson R.S., Al Rifai M., Virk H.U.H., Wang Z., Virani S., Glicksberg B.S., Lavie C.J., Krittanawong C. (2024). Artificial Intelligence in Preventive Cardiology. Prog. Cardiovasc. Dis..

[252] Bușilă C., Stuparu-Crețu M., Nechita A., Grigore C.A., Balan G. (2017). Good Glycemic Control for a Low Cardiovascular Risk in Children Suffering from Diabets. Rev. De Chim..

[253] Kanegae H., Suzuki K., Fukatani K., Ito T., Harada N., Kario K. (2020). Highly Precise Risk Prediction Model for New-onset Hypertension Using Artificial Intelligence Techniques. J. Clin. Hypertens..

[254] Islam S.M.S., Talukder A., Awal M.A., Siddiqui M.M.U., Ahamad M.M., Ahammed B., Rawal L.B., Alizadehsani R., Abawajy J., Laranjo L. (2022). Machine Learning Approaches for Predicting Hypertension and Its Associated Factors Using Population-Level Data from Three South Asian Countries. Front. Cardiovasc. Med..

[255] Oh G.C., Ko T., Kim J.-H., Lee M.H., Choi S.W., Bae Y.S., Kim K.H., Lee H.-Y. (2022). Estimation of Low-Density Lipoprotein Cholesterol Levels Using Machine Learning. Int. J. Cardiol..

[256] Wu J., Qin S., Wang J., Li J., Wang H., Li H., Chen Z., Li C., Wang J., Yuan J. (2020). Develop and Evaluate a New and Effective Approach for Predicting Dyslipidemia in Steel Workers. Front. Bioeng. Biotechnol..

[257] Correia M., Kagenaar E., van Schalkwijk D.B., Bourbon M., Gama-Carvalho M. (2021). Machine Learning Modelling of Blood Lipid Biomarkers in Familial Hypercholesterolaemia versus Polygenic/EnvironmentalDyslipidaemia. Sci. Rep..

[258] Bușilă C., Stuparu-Crețu M., Barna O., Balan G. (2017). Dyslipidemia in Children as a Risk Factor for Cardiovascular Diseases. Biotechnol. Biotechnol. Equip..

[259] Adedinsewo D.A., Pollak A.W., Phillips S.D., Smith T.L., Svatikova A., Hayes S.N., Mulvagh S.L., Norris C., Roger V.L., Noseworthy P.A. (2022). Cardiovascular Disease Screening in Women: Leveraging Artificial Intelligence and Digital Tools. Circ. Res..

[260] Tseng A.S., Thao V., Borah B.J., Attia I.Z., Medina Inojosa J., Kapa S., Carter R.E., Friedman P.A., Lopez-Jimenez F., Yao X. (2021). Cost Effectiveness of an Electrocardiographic Deep Learning Algorithm to Detect Asymptomatic Left Ventricular Dysfunction. Mayo Clin. Proc..

[261] Barry T., Farina J.M., Chao C.-J., Ayoub C., Jeong J., Patel B.N., Banerjee I., Arsanjani R. (2023). The Role of Artificial Intelligence in Echocardiography. J. Imaging.

[262] Nedadur R., Wang B., Tsang W. (2022). Artificial Intelligence for the Echocardiographic Assessment of Valvular Heart Disease. Heart.

[263] Almansouri N.E., Awe M., Rajavelu S., Jahnavi K., Shastry R., Hasan A., Hasan H., Lakkimsetti M., AlAbbasi R.K., Gutiérrez B.C. (2024). Early Diagnosis of Cardiovascular Diseases in the Era of Artificial Intelligence: An in-Depth Review. Cureus.

[264] Sengupta P.P., Shrestha S., Kagiyama N., Hamirani Y., Kulkarni H., Yanamala N., Bing R., Chin C.W.L., Pawade T.A., Messika-Zeitoun D. (2021). A Machine-Learning Framework to Identify Distinct Phenotypes of Aortic Stenosis Severity. JACC Cardiovasc. Imaging.

[265] Yang F., Chen X., Lin X., Chen X., Wang W., Liu B., Li Y., Pu H., Zhang L., Huang D. (2022). Automated Analysis of Doppler Echocardiographic Videos as a Screening Tool for Valvular Heart Diseases. JACC Cardiovasc. Imaging.

[266] Zhang J., Zhang J., Jin J., Jiang X., Yang L., Fan S., Zhang Q., Chi M. (2024). Artificial Intelligence Applied in Cardiovascular Disease: A Bibliometric and Visual Analysis. Front. Cardiovasc. Med..

[267] Zhang Q., Zheng P., Hong Z., Li L., Liu N., Bian Z., Chen X., Wu H., Zhao S. (2024). Machine Learning in Risk Prediction of Continuous Renal Replacement Therapy after Coronary Artery Bypass Grafting Surgery in Patients. Clin. Exp. Nephrol..

[268] Bivolaru S., Constantin A., Vlase C.M., Gutu C. (2023). COPD Patients’ Behaviour When Involved in the Choice of Inhaler Device. Healthcare.

[269] De Ramón Fernández A., Ruiz Fernández D., Gilart Iglesias V., Marcos Jorquera D. (2022). Analyzing the Use of Artificial Intelligence for the Management of Chronic Obstructive Pulmonary Disease (COPD). Int. J. Med. Inform..

[270] Al Namat R., Duceac L.D., Chelaru L., Dabija M.G., Guțu C., Marcu C., Popa M.V., Popa F., Bogdan Goroftei E.R., Țarcă E. (2023). Post-Coronary Artery Bypass Grafting Outcomes of Patients with/without Type-2 Diabetes Mellitus and Chronic Kidney Disease Treated with SGLT2 Inhibitor Dapagliflozin: A Single-Center Experience Analysis. Diagnostics.

[271] Moinul M., Amin S.A., Kumar P., Patil U.K., Gajbhiye A., Jha T., Gayen S. (2022). Exploring Sodium Glucose Cotransporter (SGLT2) Inhibitors with Machine Learning Approach: A Novel Hope in Anti-Diabetes Drug Discovery. J. Mol. Graph. Model..

[272] Vidal-Perez R., Grapsa J., Bouzas-Mosquera A., Fontes-Carvalho R., Vazquez-Rodriguez J.M. (2023). Current Role and Future Perspectives of Artificial Intelligence in Echocardiography. World J. Cardiol..

